# Protein Molecular Function Prediction by Bayesian Phylogenomics

**DOI:** 10.1371/journal.pcbi.0010045

**Published:** 2005-10-07

**Authors:** Barbara E Engelhardt, Michael I Jordan, Kathryn E Muratore, Steven E Brenner

**Affiliations:** 1 Department of Electrical Engineering and Computer Sciences, University of California, Berkeley, California, United States of America; 2 Department of Statistics, University of California, Berkeley, California, United States of America; 3 Department of Molecular and Cell Biology, University of California, Berkeley, California, United States of America; 4 Department of Plant and Microbial Biology, University of California, Berkeley, California, United States of America; The Institute for Genomic Research, United States of America

## Abstract

We present a statistical graphical model to infer specific molecular function for unannotated protein sequences using homology. Based on phylogenomic principles, SIFTER (Statistical Inference of Function Through Evolutionary Relationships) accurately predicts molecular function for members of a protein family given a reconciled phylogeny and available function annotations, even when the data are sparse or noisy. Our method produced specific and consistent molecular function predictions across 100 Pfam families in comparison to the Gene Ontology annotation database, BLAST, GOtcha, and Orthostrapper. We performed a more detailed exploration of functional predictions on the adenosine-5′-monophosphate/adenosine deaminase family and the lactate/malate dehydrogenase family, in the former case comparing the predictions against a gold standard set of published functional characterizations. Given function annotations for 3% of the proteins in the deaminase family, SIFTER achieves 96% accuracy in predicting molecular function for experimentally characterized proteins as reported in the literature. The accuracy of SIFTER on this dataset is a significant improvement over other currently available methods such as BLAST (75%), GeneQuiz (64%), GOtcha (89%), and Orthostrapper (11%). We also experimentally characterized the adenosine deaminase from *Plasmodium falciparum*, confirming SIFTER's prediction. The results illustrate the predictive power of exploiting a statistical model of function evolution in phylogenomic problems. A software implementation of SIFTER is available from the authors.

## Synopsis

New genome sequences continue to be published at a prodigious rate. However, unannotated sequences are of limited use to biologists. To computationally annotate a hypothetical protein for molecular function, researchers generally attempt to carry out some form of information transfer from evolutionarily related proteins. Such transfer is most successfully achieved within the context of phylogenetic relationships, exploiting the comprehensive knowledge that is available regarding molecular evolution within a given protein family. This general approach to molecular function annotation is known as phylogenomics, and it is the best method currently available for providing high-quality annotations. A drawback of phylogenomics, however, is that it is a time-consuming manual process requiring expert knowledge. In the current paper, the authors have developed a statistical approach—referred to as SIFTER (Statistical Inference of Function Through Evolutionary Relationships)—that allows phylogenomic analyses to be carried out automatically.

The authors present the results of running SIFTER on a collection of 100 protein families. They also validate their method on a specific family for which a gold standard set of experimental annotations is available. They show that SIFTER annotates 96% of the gold standard proteins correctly, outperforming popular annotation methods including BLAST-based annotation (75%), GOtcha (89%), GeneQuiz (64%), and Orthostrapper (11%). The results support the feasibility of carrying out high-quality phylogenomic analyses of entire genomes.

## Introduction

The post-genomic era has revealed the nucleic and amino acid sequences for large numbers of genes and proteins, but the rate of sequence acquisition far surpasses the rate of accurate protein function determination. Sequences that lack molecular function annotation are of limited use to researchers, so automated methods for molecular function annotation attempt to make up for this deficiency. But the large number of errors in protein function annotation propagated by automated methods reduces their reliability and utility [1–3].

Most of the well-known methods or resources for molecular function annotation, such as BLAST [4], GOFigure [5], GOtcha [6], GOblet [7], OntoBlast [8], GeneMine [9], PFUNCTIONER [10], PEDANT [11], MAGPIE [12], GeneQuiz [13], the COGs database [14], and HOVERGEN/HOBACGEN [15], rely on sequence similarity, such as a BLAST *E*-value, as an indicator of homology. A functional annotation is heuristically transferred to the query sequence based on reported functions of similar sequences.

SIFTER (Statistical Inference of Function Through Evolutionary Relationships) takes a different approach to function annotation. Phylogenetic information, if leveraged correctly, addresses many of the weaknesses of sequence-similarity-based annotation transfer [16], such as ignoring variable mutation rates [17,18]. Orthostrapper [19] and RIO [20] provide examples of methods that exploit phylogenetic information, but these methods simplify the problem by extracting pairwise comparisons from the phylogeny, and by using heuristics to convert these comparisons into annotations. SIFTER is a more thoroughgoing approach to automating phylogenomics that makes use of a statistical model of molecular function evolution to propagate all observed molecular function annotations throughout the phylogeny. Thus, SIFTER is able to leverage high-quality, specific annotations and to combine them according to the overall pattern of phylogenetic relationships among homologous proteins.

Other approaches, referred to as context methods, predict protein function using evolutionary information and protein expression and interaction data [21–26]. These methods provide predictions for functional interactions and relationships. They complement detailed predictions from SIFTER and the sequence-based approaches mentioned above, which predict features that evolve in parallel with molecular phylogenetic relationships, such as molecular function.

### Phylogenomics

Phylogenomics is a methodology for annotating the specific molecular function of a protein using the evolutionary history of that protein as captured by a phylogenetic tree [17]. Phylogenomics has been used to assign precise functional annotations to proteins encoded in a number of recently sequenced genomes [27,28] and specific protein families [29], despite being a time-consuming manual process. Phylogenomic ideas have also proven helpful for addressing general evolutionary questions, such as showing that horizontal gene transfer is much less common between bacteria and human genes than was suggested in the original publication of the human genome [30,31].

Phylogenomics applies knowledge about how molecular function evolves to improve function prediction. Specifically, phylogenomics is based on the assertion that protein function evolves in parallel with sequence [32], implying that a phylogeny based on protein sequences accurately represents how molecular function evolved for that particular set of proteins. Additionally, molecular function tends to evolve more rapidly after duplication than after speciation because there are fewer mutational constraints; thus, mutations that alter function may more easily fixate in one of the copies [33–35]. These observations give rise to the phylogenomics method, which involves building a phylogenetic tree from homologous protein sequences, marking the location of duplication events, and propagating known functions within each clade descendant from a duplication event. This produces a set of function predictions supported by the evolutionary principles outlined above.

It is broadly recognized that this method produces high-quality results for annotating proteins with specific molecular functions [16]. Three problems limit its feasibility for universal application. First, phylogenomic analysis is a labor-intensive manual process that requires significant effort from dedicated scientists. Second, the quality of the predictions depends on the expertise of the scientist performing the annotation and the quality and availability of functions for the homologous proteins. Third, phylogenomics does not provide a consistent methodology for reporting when a function has insufficient support because of sparse, conflicting, or evolutionarily distant evidence. These three problems motivate the development of a statistical methodology for phylogenomics.

### Bayesian Statistics in Biology

Bayesian methodologies have influenced computational biology for many years [36]. Bayesian methods give robust, consistent means of incorporating evidence, even when it is sparse, and enable different types of evidence to be integrated in a meaningful way. The specific inference method we developed for phylogenomics (see Materials and Methods) is based on the general formalism of probabilistic graphical models [37]. It has roots in the peeling methods for pedigree analysis [38,39], and later in maximum likelihood methods for reconstructing phylogenies [40]. We have chosen to take a Bayesian approach to calculating the posterior probabilities of each molecular function for each protein, addressing the uncertainty in the unobserved variables in the phylogeny using Bayesian inference but assuming the phylogeny is known. This is in contrast to the bootstrap approach as taken in RIO and Orthostrapper, which calculate bootstrapped confidence values representing the percentage of trees in which two proteins are orthologous. These methods address the uncertainty of the phylogeny structure (using a frequentist approach), but assume the values of the unobserved variables are known given the phylogeny.

Three properties of the Bayesian approach make it uniquely suited to molecular function prediction. First, Bayesian inference exploits all of the available observations, a feature that proves to be essential in this inherently observation-sparse problem. Second, the constraints of phylogenomics—that function mutation tends to occur after a duplication event or that function evolution proceeds parsimoniously—are imposed as prior biases, not as hard constraints. This provides a degree of robustness to assumptions that is important in a biological context. Third, Bayesian methods also tend to be robust to errors in the data. This is critical in our setting, not only because of existing errors in functional annotations, but also because phylogeny reconstruction and reconciliation often imperfectly reflect evolutionary history.

The current instantiation of SIFTER uses Bayesian inference to combine all molecular function evidence within a single phylogenetic tree, using an evolutionary model of molecular function. A fully Bayesian approach to phylogenomics would integrate over all sources of uncertainty in the function annotation problem, including uncertainty in the phylogeny and its reconciliation, and uncertainty in the evolutionary model for molecular function. It is important to be clear at the outset that the current instantiation of SIFTER stops well short of full Bayesian integration. Rather, we have focused on a key inferential problem that is readily treated with Bayesian methods and is not accommodated by current tools in the literature—that of combining all of the evidence within a single inferred tree using probabilistic methods. Technically, this limited use of the Bayesian formalism is referred to as “empirical Bayes” [41].

Extensions to a more fully Bayesian methodology are readily contemplated; for example, we could use techniques such as those used by MrBayes [42] to integrate across phylogenies. In preliminary investigation of the robustness of SIFTER, however, we have performed bootstrap resampling of the reconciled trees and found little variation in our results across bootstrap samples (results will be detailed elsewhere). This suggests that much of the gain in using Bayesian methods may accrue at the level of inference within a single tree, a suggestion supported by the results that we present here comparing SIFTER to Orthostrapper, which is similar to SIFTER in its use of phylogenomic concepts but differs critically in that it does not integrate evidence within trees.

### Molecular Function Annotations

All automated function annotation methods require a vocabulary of molecular function names, whether the names are from the set of Enzyme Commission (EC) numbers, Gene Ontology (GO) molecular function names [43], or words derived from existing manual annotations (e.g., Swiss-Prot functional descriptions). In our method, we currently use the well-curated molecular function ontology from GO, which provides annotations for many proteins in Swiss-Prot and TrEMBL. Each annotation in the GO annotation (GOA) database [44] includes an evidence code, which describes how the annotation was determined. These codes include IDA (inferred by direct assay), IMP (inferred by mutant phenotype), and IEA (inferred by electronic annotation), and they can be used to crudely estimate the reliability of the reported function for a protein.

### SIFTER Approach

SIFTER builds upon phylogenomics by employing statistical inference algorithms to propagate available function annotations within a phylogeny, instead of relying on manual inference, as fully described in Materials and Methods. Statistical inference requires a probabilistic model of how the character states (in this case, molecular function) evolve; to this end, we constructed a model of molecular function evolution to infer function in a reconciled phylogeny. Our model takes into account evidence of varying quality and computes a posterior probability for every possible molecular function (from the set of GO molecular function terms) for each protein in the phylogeny, including ancestor proteins. In our model, each molecular function may evolve from any other function, and a protein's function may evolve more rapidly after duplication events than after speciation events.

A “duplication event” captures a single instance of a gene duplicating into divergent copies of that gene within a single genome; a “speciation event” captures a single instance of a gene in an ancestral species evolving into divergent copies of a gene in distinct genomes of different species. Each of the internal nodes of a phylogeny represents one of these two events, although a standard phylogeny does not distinguish between the two. The reconciled phylogeny for a protein family, which discriminates duplication events and speciation events [45,46], specifies the tree-structured graphical model used in inference. In this work, we do not estimate the locations of gene deletion, as it can be difficult to differentiate gene deletion from partial sampling of genes within a particular family.

The available, or observed, function annotations, associated with individual proteins at the leaves of the phylogeny, are propagated towards the root of the phylogeny and then propagated back out to the leaves of the phylogeny, based on a set of update equations defined by the model of function evolution. The result of the inference procedure is a posterior probability of each molecular function for every node in the tree (including the leaves), conditioned on the set of observed functions. The posterior probabilities at each node do not actually select a unique functional annotation for that node, so functional predictions may be selected using a decision rule based on the posterior probabilities of all of the molecular functions. This procedure gives statistical meaning to the phylogenomic notion of propagating functional annotations throughout each clade descendant from a molecular function mutation event. We do not require that the mutation event coincide with a duplication event.

The inference algorithm used in SIFTER has linear complexity in the size of the tree and thus is viable for large families. The complexity of SIFTER is exponential in the number of possible molecular functions in a family, owing to the fact that we compute posterior probabilities for all possible subsets of functions. In the families that we studied, the number of functions was small and this computation was not rate-limiting; in general, however, it may be necessary to restrict the computation to smaller collections of subsets. The rate-limiting step of applying SIFTER is phylogeny reconstruction; a full-genome analysis, given limited computational resources, might use lower-quality or precomputed phylogenies along with bootstrapping, or a subset of closely related species for the larger protein families. We found that lower-quality trees do not significantly diminish the quality of the results (results will be detailed elsewhere).

In this report, we use only GO IDA- and IMP-derived annotations as observations for SIFTER, because of the high error rate and contradictions in the non-experimental annotations (i.e., all annotation types besides IDA and IMP). However, SIFTER can also incorporate other types of annotations, weighted according to their reliability.

## Results

We first present results for SIFTER's performance on a large set of proteins to show general trends in prediction and to evaluate the scalability of SIFTER. We then present results for a single protein family with a gold standard set of function characterizations to evaluate prediction quality in detail. We also describe results for the lactate/malate dehydrogenase family, although it does not have a gold standard dataset. The decisive benefit of a statistical approach to phylogenomics is evidenced on each of these different datasets.

### Results for 100 Pfam Families

To evaluate the scalability, applicability, and relative performance of SIFTER, we predicted molecular function for proteins from 100 protein families available in Pfam [47], using experimental annotations from the GOA database as evidence. On this broad set of proteins, there are no gold standard functional annotations to which we can meaningfully compare SIFTER's predictions. Instead, we compared SIFTER's predictions to the non-experimental annotations from the GOA database, GOtcha [6], Orthostrapper [19], and BLAST-based predictions [4] in order to measure trends of agreement and compatibility.

For each family in our 100-family dataset, we ran SIFTER on the associated reconciled tree with the experimental annotations (IDA and IMP) from the GOA database. SIFTER produced a total of 23,514 function predictions; we selected the subset of 18,736 that had non-experimental annotations from the GOA database and applied BLASTC, GOtcha, and Orthostrapper to this set (Table 1). We did not compare annotations for experimentally characterized proteins, as those observations were used for inference in SIFTER and Orthostrapper. We compared SIFTER's predictions against non-experimental annotations from the GOA database and function predictions from the other methods. In addition to considering identical GO terms, we also considered terms on the same path to the root of the GO directed acyclic graph (DAG); we call the latter “compatible” annotations because although they are not identical they do not disagree, even though one may be much more specific than the other (and possibly incorrect).

**Table 1:**
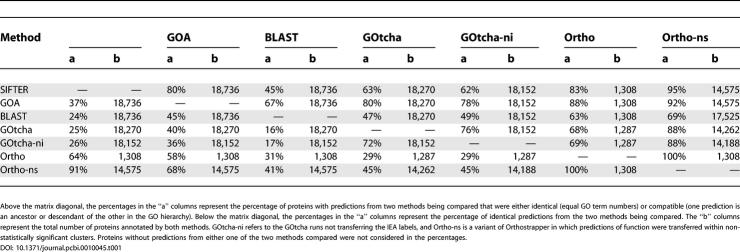
Comparison of Predicted Annotations on 18,736 Proteins from 100 Pfam Families

We chose these 100 families to meet one of the following two criteria: (1) greater than 10% proteins with experimental annotations (and more than 25 proteins), or (2) more than nine experimental annotations. Families with fewer than two incompatible experimental GO functions were excluded. The families had an average of 235 proteins, ranging from 25 to 1,116 proteins. On average, 3.3% of the proteins in a family had IDA annotations, and 0.4% had IMP annotations. Both SIFTER and Orthostrapper relied on this particularly sparse dataset for inference; evaluative techniques involving the removal of any of these annotations from inference tended to trivialize the results (e.g., removing a lone experimental annotation for a particular function did not enable that function prediction for homologous proteins). Selecting well-annotated families via these criteria assists SIFTER, but it should also enhance the performance of all of the function transfer methods evaluated here. Note also that SIFTER does not require this level of annotation accuracy to be effective, as discussed below. Finally, it is important to note that many of the IEA annotations from the GOA database may come from one of the assessed methods, so we can expect consistency to be quite high.

Of the 8,501 SIFTER predictions that were either identical or incompatible to the GO non-experimental annotations, 83.1% were identical. The average percentage of identical function predictions by family was 82.9%, signifying that the size of the family does not appear to impact this percentage. The median identity by family was 90.7%, and the mode was 100% (representing 25 families). The minimum identity was 14.4% (Pfam family PF00536). We estimate that 38 of the families contained non-enzyme proteins, and we found no difference in the identity percentage of SIFTER on enzyme families versus non-enzyme families. Similarly, the total number of functional annotations used as observations in SIFTER does not appear to impact the identity percentage (although percentage of proteins with annotations does appear to impact identity percentage). These data suggest that a large percentage of incompatibility is concentrated within a few families. It is not entirely clear what property of those families contributes to the greater incompatibility; it may reflect how well studied the families are relative to the number of proteins in the family.

#### Annotation rates.

Not all of the annotation methods predict functions for 100% of the proteins. Indeed, as shown in Table 2, Orthostrapper was able to annotate only 7% of the proteins. In an effort to improve the annotation rate, we implemented a variant of Orthostrapper (referred to as “Ortho-ns” in the tables) in which functional annotations were transferred within non-significant orthologous clusters. The nominal mode of operation of Orthostrapper is to transfer function within “statistically significant clusters,” defined as those in which proteins are transitively orthologous with one another in at least 75% of the phylogenies built from a bootstrapped alignment. For Ortho-ns we lowered the criterion from 75% to 0.1%. This yielded an annotation rate of 77%, significantly higher than Orthostrapper, but still well short of the rate of the other methods.

**Table 2:**
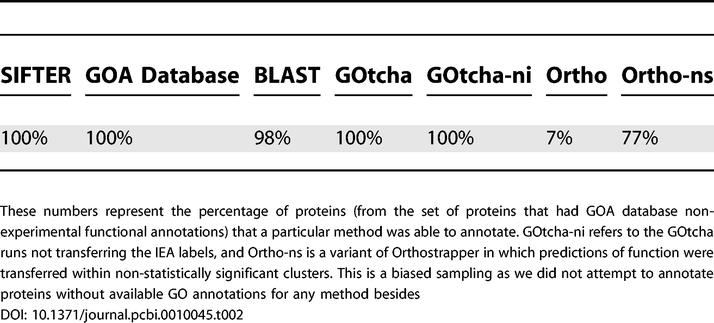
Prediction Coverage on 18,736 Proteins from 100 Pfam Families

The percentage of Orthostrapper predictions that were identical or compatible with the non-experimental GOA database function annotations in the 100-family dataset was 88%, but only 7% of proteins received Orthostrapper predictions (Table 2). When function is transferred within non-statistically significant clusters, agreement or compatibility goes to 92% for the 77% of proteins that now receive predictions.

The difficulties encountered by Orthostrapper arise from the small number of proteins that are placed in statistically supported clusters, and the lack of annotations in these clusters. The latter limits the usefulness of the method to protein families with a high percentage of known protein functions, or to observed annotations with a low error rate. These results highlight the impact of the modeling choices in SIFTER and Orthostrapper. SIFTER uses Bayesian inference in a single phylogeny, addressing uncertainty in the ancestral variables in the phylogeny but presently not addressing uncertainty in the phylogeny itself. In contrast, Orthostrapper's approach of bootstrapped orthology addresses uncertainty in the phylogeny, but neglects uncertainty in the ancestral variables. Our results indicate that the gains to be realized by treating uncertainty within a tree may outweigh those to be realized by incorporating uncertainty among trees, but it would certainly be of interest to implement a more fully Bayesian version of SIFTER that accounts for both sources of uncertainty.

#### Prediction comparisons.

We compared SIFTER's prediction (the function with the single highest posterior probability) to the top-ranked prediction from BLAST-based methods, the top-ranked prediction from GOtcha, the unranked set of non-experimental terms from the GOA database, and unranked Orthostrapper predictions. On this broad set of proteins, SIFTER's predictions were compatible or identical with the non-experimental annotations from the GOA database for 80% of the predictions, while 67% of BLAST-based predictions, 80% of GOtcha predictions, and 78% of GOtcha-ni predictions were compatible or identical to the non-experimental GOA database annotations. It is not entirely clear what these numbers represent, in particular because some unknowable fraction of the IEA annotations in the GOA database were derived using these or related methods. Orthostrapper predictions achieved 88% (Ortho) and 92% (Ortho-ns) compatibility or identity with the GOA database, but because of the small percentage of proteins receiving predictions using Orthostrapper, the absolute number of compatible or identical annotations is much lower. All pairwise comparison data are in Table 1.

The number of incompatible annotations is noteworthy: exact term agreement ranges from 16% to 91%, and the percentage of compatible or identical terms ranges from 45% to 95%. Collectively the methods must be producing a large number of incorrect annotations as evidenced by the high percentage of disagreement in predictions. It appears that there is no gold standard for comparison in the case of electronic annotation methods other than experimental characterization.

### Adenosine-5′-Monophosphate/Adenosine Deaminase: A Gold Standard Family

We selected a well-characterized protein family, the adenosine-5′-monophosphate (AMP)/adenosine deaminase family, for evaluation of SIFTER's predictions against a gold standard set of function annotations. We assessed these using experimental annotations that we manually identified in the literature, accepting only first-hand experimental results that were successful in unambiguously characterizing the specific chemical reaction in question. References are provided in Dataset S1 for each protein characterized in this way. The “accuracy” percentages presented here reflect the product of the percentage of proteins that received a prediction and, of those, the percentage that were “correct,” i.e., had the same GO terms as the gold standard test set.

**Figure 1:**
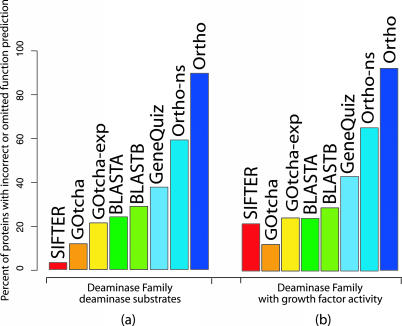
Percentage of Proteins with Incorrect or Omitted Molecular Function Prediction of the AMP/Adenosine Deaminase Family, Assessed on a Gold Standard Test Set Results for SIFTER, BLASTA (the most significant non-identity annotated sequence), BLASTB (the most significant non-identity sequence), GeneQuiz, GOtcha, GOtcha-exp (only experimental GO annotations used), Orthostrapper (significant clusters), and Orthostrapper-ns (non-significant clusters). The gold standard test set was manually compiled based on a literature search. All percentages are of true positives relative to the test set. (A) Results for discrimination between just the three deaminase substrates, as a percentage of the 28 possible correct functions. (B) Results for discrimination between the three deaminase substrates plus the additional growth factor domain, as a percentage of the 36 possible correct functions; for BLAST, GeneQuiz, Orthostrapper, and Orthostrapper-ns, we required the transferred annotation to contain both functions; for SIFTER, GOtcha, and GOtcha-exp we required that the two correct functions have the two highest ranking posterior probabilities or scores.

The AMP/adenosine deaminase Pfam family contains 128 proteins. Based on five proteins with experimental annotations from the GOA database, we ran SIFTER to make predictions for the remaining 123 proteins. Of these remaining proteins, 28 had experimental characterizations found by the manual literature search. SIFTER achieved 96% accuracy (27 of 28) for predicting a correct function against this gold standard dataset. SIFTER performed better than BLAST, GeneQuiz, GOtcha, GOtcha-exp (GOtcha transferring only experimental GO annotations), and Orthostrapper (75%, 64%, 89%, 79%, and 11% accuracy, respectively). The comparative results are summarized in Figure 1A. The complete data for these analyses are available in Dataset S1.

**Figure 2:**
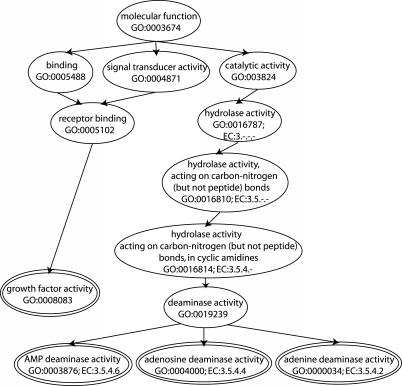
Gene Ontology Hierarchy Section Representing the Functions Associated with the Three Substrate Specificities Found in the AMP/Adenosine Deaminase Pfam Family, and the Growth Factor Activity Associated with a Few Members of the Family Double ovals represent the four functions, none of which are compatible, corresponding to the random variables associated with the random vector used for inference in SIFTER.

The general role of the AMP/adenosine deaminase proteins is to remove an amine group from the purine base of the substrate. The AMP/adenosine deaminase family has four GO functions associated with member proteins (Figure 2). Adenine deaminase (GO:0000034; EC:3.5.4.2) catalyzes the hydrolytic deamination of adenine to ammonia and hypoxanthine, which is a metabolic nitrogen source [48]. Adenosine deaminase (GO:0004000; EC:3.5.4.4) modifies post-transcriptional RNA, converting adenosine to inosine, resulting in a protein with a sequence different from that coded in the genome by the standard codon table [49]. A mutation in the adenosine deaminase protein in *Homo sapiens* results in one form of severe combined immune deficiency syndrome [50]. AMP deaminase (GO:0003876; EC:3.5.4.6) converts AMP into inosine-5′-monophosphate and ammonia, and is critical in carbohydrate metabolism [51]. A subset of the adenosine deaminase proteins include multi-domain proteins, in which the additional domain is associated with growth factor activity (GO:0008083, not an enzyme function) (e.g., [52]), and we discuss this additional domain later in Results. Given the functionally important and distinct roles of these related proteins, being able to differentiate at the level of substrate specificity is a critical aspect of function prediction.

**Figure 3:**
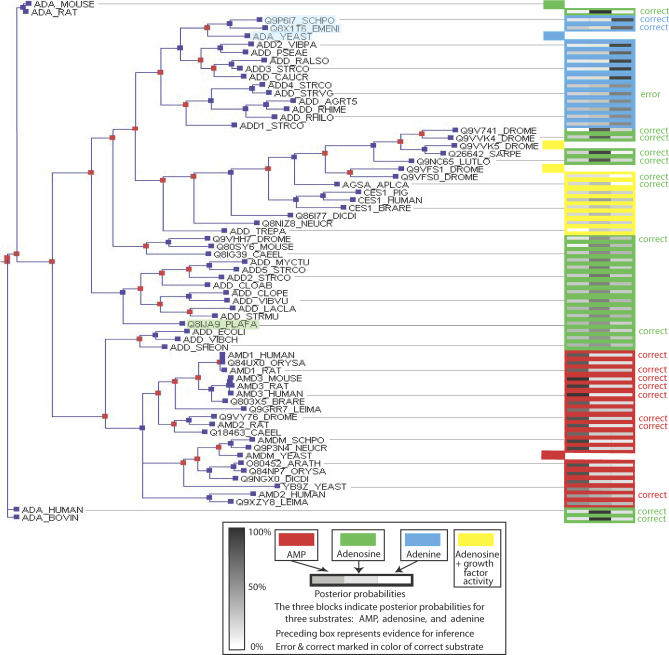
Results for Pruned Version of the AMP/Adenosine Deaminase Family The reconciled phylogeny used in inference is shown, along with inferential results (both the posterior probabilities for the deaminase substrates and the function prediction based on the maximum posterior probability). Eight of the proteins in this tree were annotated with growth factor activity, with the second highest probability being adenosine deaminase. The function observations used for inference are denoted by filled boxes to the left of the column with the posterior probabilities. For each substrate specificity that arises, a single edge in the phylogeny identifies a possible location for that mutation. The highlighted sequences are discussed in the text. The blue vertices represent speciation events and the red vertices represent duplication events. The tree was rendered using ATV software, version 1.92 [68].

The prediction results, a subset of which are shown in Figure 3, illustrate how statistical inference captures the phylogenomic principle of propagating function throughout clades descendant from duplication or speciation events where a function mutation may have occurred. The posterior probability for each annotation provides a measure of confidence in each possible function annotation, based on the model of function evolution and the reported functions for the five proteins with GOA database experimental annotations. The confidence for a particular function annotation tends to drop as the tree-based distance from the closest observation of that function increases.

**Figure 4:**
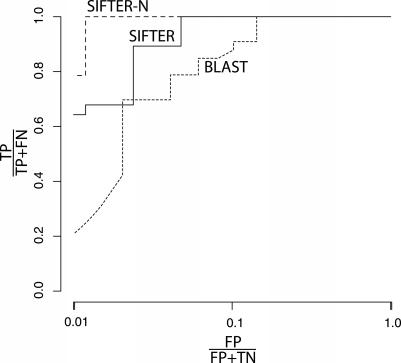
ROC Plots for the AMP/Adenosine Deaminase Family Functional Predictions from BLASTC, SIFTER, and SIFTER-N (Normalized) These ROC curves were computed over the 28 proteins in the test set for the deaminase family. This figure presents the ROC plot for both the posterior probabilities produced by SIFTER (and normalized for SIFTER-N) and the *E*-value significance scores from BLASTC, where they are used to annotate proteins, selecting between deaminase substrates AMP, adenine, and adenosine. The false positive axis is scaled logarithmically to focus on true positive percentages when the percentage of false positives is low. FN, false negative; FP, false positive; TN, true negative; TP, true positive.

An alternate method to evaluate prediction accuracy is the receiver operating characteristic (ROC) plot. Figure 4 shows the ROC plot for discriminating the three deaminase substrates (AMP, adenine, and adenosine) using the posterior probabilities from SIFTER, with 64% coverage (i.e., percentage of proteins annotated correctly) at 1% false positives. We logarithmically scaled the false positive axis to focus on true positive percentages when the percentage of false positives is low. The purpose of the ROC plot here is to show that a user-specified cutoff value (based on percentage of false positives at that cutoff) may be used to identify when a functional prediction should not be made for a particular protein. With such a cutoff, we can identify proteins for which the posterior probability of every molecular function is too low to support a prediction.

Unmodified posterior probabilities allow us to assess the quality of a functional prediction across a family. When considering subsets of functional predictions, however, the maximum posterior for a protein may be small compared to the maximum posterior for other proteins, but we still would like to select this functional assignment because the other posteriors for this protein are smaller still. This can be achieved by normalizing the posteriors for a given protein across the subset of functional assignments of interest. For discriminating the three deaminase substrates, Figure 4 shows the results using renormalized posteriors as the curve labeled SIFTER-N. SIFTER-N achieved 79% coverage at 1% false positives, showing that the correct function has the dominant posterior probability for nearly all proteins. This result implies that choosing a single cutoff value as a decision rule for the unmodified posteriors may not be appropriate for certain biological questions.

#### Comparison with existing methods.

We compared SIFTER's predictions in this family to four available protein function annotation methods: BLAST (in two approaches called BLASTA and BLASTB, as described in Materials and Methods), GeneQuiz, GOtcha, and Orthostrapper. The complete summary results are shown in Figure 1A.

On this gold standard annotation dataset, SIFTER predictions were more accurate than the alternative methods. Caveats must be mentioned for two of the methods. We ran GOtcha in two different ways on this dataset, detailed in Materials and Methods. GOtcha-exp, which includes only experimental GO annotations for each GOtcha prediction, allows GOtcha access to the same annotation data that SIFTER and Orthostrapper use for inference, creating a more comparable set of predictions. For GOtcha-exp, of the 22 correct annotations, nine were ties between the correct substrate and an incorrect substrate that we resolved in favor of the correct substrate. Orthostrapper was inhibited by failing to annotate some proportion of the proteins with functional characterizations, as in the 100-family dataset. Orthostrapper provided correct annotations for 11% of the proteins; this is because it annotated three correctly, and failed to annotate 25. Orthostrapper using clusters without statistical support (Ortho-ns) provided correct function prediction for 39% of the proteins, correctly annotating 11 of the 28 characterized proteins and omitting the remainder. All of the other methods annotated all of the proteins in the gold standard test set.

If we accept compatible annotations, the accuracy for BLASTA improves by a single protein (Q9VHH7) to 79% (22 of 28), and BLASTB results remain the same (see Materials and Methods for these protocol definitions). To evaluate BLAST in a more sophisticated way, we applied the BLASTC protocol (see Materials and Methods) to find the *E*-value of the top non-identity hit with an annotation in our selected subset of functions (i.e., adenosine deaminase, adenine deaminase, and AMP deaminase). Although BLAST is not generally applied to molecular function prediction in this way, this comparison enables a more critical assessment of SIFTER. These *E*-values were used to plot BLAST on the ROC plot (Figure 4, BLAST label). BLASTC achieved 21% coverage at 1% false positives, and is visibly inferior to the coverage provided by both SIFTER and SIFTER-N.

#### Multi-domain proteins.

The deaminase results focus on a single homologous protein domain that deaminates three possible substrates (AMP, adenosine, and adenine). A few of the proteins in this Pfam family have an additional N-terminal domain. This extra domain (PB003508) has growth factor activity (GO:0008083, not an enzyme function), while the AMP/adenosine binding domain (PF00962) has adenosine deaminase function. We built the phylogeny for this family using only the common AMP/adenosine binding domain; the functional annotations, however, are affiliated with the entire protein sequence. Because the phylogenomic model currently does not explicitly address domain fusion events, we did not consider the molecular function associated with the additional domain in the analyses described thus far.

We reevaluated the results, requiring, where appropriate, growth factor activity to be in the transferred functional annotation (for BLAST, Orthostrapper, and GeneQuiz), or requiring “adenosine deaminase” and “growth factor activity” to have the two highest rankings (ranked by posterior probabilities for SIFTER, or scores for GOtcha and GOtcha-exp). This provided a total of 36 molecular functions to be annotated on the 28 proteins. When evaluating the ability of methods to also correctly annotate this additional role, the accuracy for every method decreases or remains consistent. SIFTER achieved 78% accuracy (28 of 36), while BLAST achieved 75% accuracy (27 of 36), and GeneQuiz achieved 58% accuracy (21 of 36). GOtcha predictions achieved 89% accuracy in the multi-domain setting (32 of 36), and GOtcha-exp achieved 75% (27 out of 36). Of the 11% of proteins that Orthostrapper annotated, none were in the set of proteins with growth factor (so overall accuracy is 8% of the 36 functions to annotate); considering non-statistically significant annotations, Orthostrapper (Ortho-ns) achieved 35% (58% accuracy for 61% of proteins annotated). These results are summarized in Figure 1B. This degradation trend in prediction quality highlights a problem with function annotation methods and their application to multifunction or multi-domain proteins [16,53]. SIFTER in particular appears prone to this degradation, which may be addressed in part by a more problem-specific decision rule that selects function predictions from posterior probabilities, although ultimately the statistical model for SIFTER could explicitly take protein domain architecture into account.

#### SIFTER prediction experimentally confirmed.

**Figure 5:**
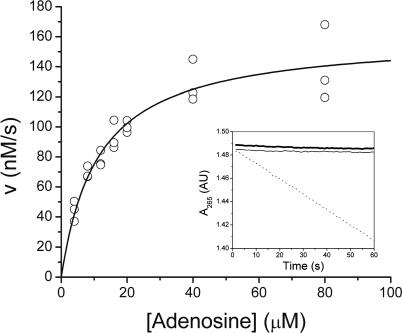
The Dependence of the Rate of Deamination of Adenosine upon Substrate Concentration with 17 nM Q8IJA9_PLAFA The open circles are individual data points, while the solid line is the fit of the data to Equation 1. The inset shows raw data for the deamination of three substrates by Q8IJA9_PLAFA as detected by loss of absorbance at 265 nm. The bold, thin, and dashed lines are data for 100 μM adenine, AMP, and adenosine, respectively. The reactions with adenine and AMP contained 860 nM enzyme, while the assay containing adenosine had only 17 nM enzyme. Reaction conditions for all assays were 25 °C in 50 mM potassium phosphate (pH 7.4).

We experimentally characterized the substrate specificity of a deaminase (Q8IJA9) from the human malarial parasite, *Plasmodium falciparum*. SIFTER predicted that the preferred substrate for this enzyme is adenosine. SIFTER also predicted that the enzyme would not catalyze reactions in which AMP or adenine is the substrate. Saturation kinetics were evaluated by fitting the data to the Michaelis–Menten equation:


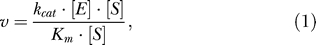


where *E* is the concentration of enzyme and *S* is the concentration of substrate. Kinetic analysis proves that this deaminase does, in fact, exhibit activity towards adenosine with a *k_cat_* of 9.3 ± 0.5 s^−1^ and a *K_m_* of 11 ± 2 μM (Figure 5). No activity was detected with either AMP or adenine at enzyme concentrations up to 860 nM (Figure 5, inset). Since the *k_cat_*/*K_m_* values for AMP and adenine are less than 10 M^−1^ s^−1^, this enzyme shows a preference for adenosine by at least five orders of magnitude.

### Lactate/Malate Dehydrogenase Family

A second family we chose for detailed analysis and validation is the lactate/malate dehydrogenase family. We used the Pfam family PF00056, representing the NAD^+^/NADP^+^ binding domain of this family of proteins. This Pfam family contains 605 proteins, 34 of which have function annotations supported by experimental evidence in the GOA database or in literature references; these 34 were used as evidence for SIFTER.

There are three GO functions associated with proteins in this family. L-lactate dehydrogenase (L-LDH) (GO:0004459; EC:1.1.1.27) catalyzes the final step in anaerobic glycolysis, converting L-lactate to pyruvate and oxidizing NADH [54]. L-malate dehydrogenase (L-MDH) NAD^+^ (GO:0030060; EC:1.1.1.37) and MDH NADP^+^ (GO:0046554; EC:1.1.1.83) catalyze the reversible reaction of malate to oxaloacetate using either NADH or NADPH as a reductant [55]. Although the detailed analysis will be described elsewhere, two aspects of the analysis illuminate the power of SIFTER and are discussed briefly here.

#### Rapid function mutation.

An interesting property of the SIFTER analysis is that it reports three instances of convergent evolution in the dehydrogenase family, all of which are supported in the literature, but only one of which is explicitly documented as convergent evolution [56]. One type of convergent evolution, homoplasy, occurs when a substrate specificity arises from mutations at multiple locations independently in a single phylogenetic tree. Convergent evolution demonstrates that small changes in sequence space do not necessarily correspond to small changes in function space. In particular, when substrate specificity is correlated with a small number of amino acids, molecular function may evolve rapidly. Standard phylogenomics and sequence-based annotation transfer methods are less effective at reporting convergent evolution due to rapid function mutation because of the built-in assumption that sequence and molecular function evolve parsimoniously in parallel. The impact of a particular function annotation associated with a large number of proteins within a significantly short evolutionary distance of the query protein does not allow a small clade with a different function prediction to emerge, since lack of evidence and small evolutionary distances are often insufficient to support a function mutation. By making this assumption probabilistic, SIFTER was able to report three instances of convergent evolution within this family, illustrating another benefit of approaching the problem using Bayesian methods (details provided elsewhere).

#### SIFTER predictions are specific.

Although there is no gold standard dataset for this family of proteins, based on a manual literature search we gathered 421 proteins in this family that scientists have non-experimentally annotated with a specific function, including substrate and cofactor. Our comparison metric is consistency, or the percentage of protein predictions that are identical to the set of 421 available (although non-experimental) annotations. It appears that the task of discriminating the substrates of this enzyme, i.e., predicting LDH or MDH, is not a difficult one, as all of the methods achieve a high consistency: SIFTER achieves 97% consistency, BLASTA achieves 93% consistency, GeneQuiz achieves 98% consistency, and GOtcha achieves 95% consistency with a set of non-experimental annotations. The methods mentioned here made predictions for all of the 421 proteins.

When we changed the task to include discriminating function at the cofactor level (i.e., predicting one of L-LDH, MDH NAD^+^, and MDH NADP^+^, so predicting LDH or MDH is inconsistent), the prediction task became more difficult. On this task, BLASTA consistency drops to 32%, GeneQuiz consistency drops to 68%, and GOtcha consistency drops to 73%. SIFTER's consistency, however, drops only slightly, to 95%, on this set of 421 proteins. One of the primary advantages of SIFTER for scientists is the ability to produce specific function annotations, which was originally a motivation for performing a manual phylogenomic analysis. Often substrate specificity or cofactor changes the biological role of a protein significantly, as in this case and in the deaminase family; being able to differentiate between protein functions with greater precision facilitates characterization and allows subtle but significant functional distinctions to be made.

This point can also be illustrated on a larger scale using the 100-family dataset. For each compatible (but not identical) pair of predictions, we checked which of the two predictions was more specific in the GO DAG. The results are shown in Table 3. On this set of 18,736 proteins, SIFTER predictions were more specific than BLAST, GOtcha, or Orthostrapper predictions at rates of 95%–100%. GOtcha made particularly general predictions, never having more than 35% of its predictions more specific than other methods. This reflects the tendency of the scoring metric in GOtcha to give higher weights to less specific function terms. Although we can make no claim regarding the correctness of these predictions because this dataset is not a gold standard, it is clear from these data that for a diverse set of protein families, the predictions produced by SIFTER are more specific than those produced by these competitive methods.

**Table 3:**
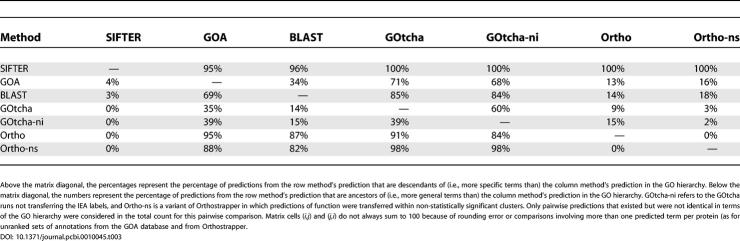
Comparison of Compatible (but Not Identical) Predicted Annotations on 18,736 Proteins from 100 Pfam Families

## Discussion

Annotation of protein function through computational techniques relies on many error-prone steps and incomplete function descriptions; SIFTER is no different than other methods in this regard. But a significant component of SIFTER is a statistical model of how protein function evolves. Devos and Valencia postulate that “the construction of a complete description of function requires extensive knowledge of the evolution of protein function that is not yet available” [57]. Although the naive model proposed here for molecular function evolution is too simple to represent how function evolves in detail, the quality of the predictions implies that it is a critical first step to building a complete statistical model that accurately captures much of protein function evolution, and has broad predictive power.

### Molecular Function Evolution

The accuracy of SIFTER's results lets us revisit the assumptions of phylogenomics with an eye towards lessons about molecular function evolution. The improvement obtained by using a tree-structured evolutionary history and evolutionary distance, as in SIFTER, versus a measure of evolutionary distance alone, as in BLAST, GOtcha, or GeneQuiz, implies that the information in evolutionary tree structure corrects the systematic errors inherent in pairwise distance methods [17] and goes much further in exploiting the parallel sequence-based tree structure to incorporate sparse data robustly. While the quality of the phylogenetic tree impacts the function predictions, bootstrap resampling of the reconciled trees illustrates that this impact is limited (results will be detailed elsewhere). Nonetheless, it would be useful to extend this analysis to a more fully Bayesian approach that integrates over reconciled phylogenies so that the method is more robust to choices of phylogeny reconstruction and reconciliation methods.

### Specific Function Annotation

Comparing SIFTER to BLAST, GeneQuiz, and GOtcha at the cofactor level for the dehydrogenase family (95% consistency versus 32%, 68%, and 73%, respectively) exemplifies the power of SIFTER over other methods for specific function prediction, and the results from the 100-family dataset lend further strength to this comparison. The difference in consistency of BLAST predictions for general substrate discrimination versus specific cofactor discrimination (61% difference) reflects the disparity between the availability of general function descriptions and specific function descriptions evolutionarily proximate to each query protein. By employing phylogenomic principles, SIFTER leveraged evolutionarily distant function observations, incorporating more specific but sparser annotations and enabling SIFTER to make specific function predictions across an entire family. BLAST, GeneQuiz, and GOtcha were limited in their ability to detail molecular function at the cofactor level because of the relative sparsity of functions reported at the cofactor level.

### Multi-Domain Proteins

A single protein sequence may contain multiple domains with several functions. There are many cases of individual domains in multi-domain proteins assuming a diverse set of functions, depending on the adjoined domains (e.g., [58]). As illustrated here, phylogenomics and models of molecular function evolution tend to lose predictive power when an additional distantly related function appears (e.g., a large path distance in the GO DAG) [16]. Because this is a relatively rare event, few models based on protein sequence exist to describe these distant functional changes (a notable exception is [59]). A more complex model including domain fusion events would improve the accuracy of SIFTER for many protein families.

### Availability of High-Quality Function Data

The sparsity of reliable data is inherent to the task of predicting protein function. In the case of the 100-family dataset, 3.7% of proteins (on average) had experimental function annotations; in the AMP/adenosine deaminase family, 2.6% of proteins had experimental function annotations. Despite this sparseness, SIFTER achieves 96% accuracy in predicting function for homologous proteins for the latter gold standard dataset. Relying exclusively on evidence derived from experimental assays ensured that the quality of the annotations was high. For the AMP/adenosine family in Pfam, there were 348 non-experimental GO annotations (for 127 of the proteins) versus three proteins with IDA (inferred from direct assay) annotations and two proteins with IMP (inferred from mutant phenotype) annotations.

There are methods of extracting annotations from literature (e.g., [60,61]) and other sources of function annotations, such as EC numbers. SIFTER can be readily modified to incorporate these alternative sources of annotations. Based on our results, it appears that SIFTER makes a prediction for a query protein at least as often as BLAST searches do. Our ongoing work focuses on quantifying this transfer rate on a genomic scale. If the posterior probabilities for the small number of specific functional terms produced by SIFTER are propagated toward the root term of the GO DAG, we have posterior probabilities for each molecular function term between the most specific and most general. We can then annotate each protein with either the most specific function prediction available at a certain confidence level or all functions with posterior probabilities above a certain cutoff.

SIFTER's primary role may be to reliably predict protein function for many of the Pfam families or more generic sets of homologous proteins. The argument can be made that no automated function annotation method should be used in some of these cases because the data within a family are too sparse to support annotation transfer. Thus, a second role for SIFTER may be to quantify the reliability of function transfer in under-annotated sets of homologous proteins, by using the posterior probabilities as a measure of confidence in annotation transfer. A third role may be to select targets for functional assays so as to provide maximum coverage based on function transfer for automated annotation techniques. Because of its Bayesian foundations, SIFTER is uniquely qualified to address these alternate questions in a quantifiable and robust way.

Molecular function predictions cannot replace direct experimental evidence for producing flawless function annotations [62]. However, computational methods for functional annotation are being called upon to fill the gap between sequence availability and functional characterization. Unfortunately, large-scale automated methods for function annotation have resulted in widespread annotation errors that reside in current databases [2,18,57,63–65]. These errors impede the progress of experimental studies by providing imprecise or incorrect molecular functions, with little indication of confidence, and minimal recourse to trace the history and origin of that function prediction. The methodology presented here aims to produce high-quality, precise, and traceable sets of possible functions for a protein with a meaningful measure of the reliability of the annotation, thereby facilitating experimental assays of molecular function and inhibiting the propagation of incorrect annotations. SIFTER is unique among function prediction methods in that it exploits phylogenomic information to infer function using formal Bayesian methods. SIFTER's prediction results, as presented here and compared with results from popular methods of function annotation, illustrate the potency and potential of exploiting evolutionary information through a statistical model of molecular function evolution.

## Materials and Methods

In this section we first present the modeling, algorithmic, and implementational choices that were made in SIFTER. We then turn to a discussion of the methods that we chose for empirical comparisons. Finally, we present the protocol followed for the deaminase activity assays.

### SIFTER model.

In classical phylogenetic analysis, probabilistic methods are used to model the evolution of characters (e.g., nucleotides and amino acids) along the branches of a phylogenetic tree [40] and to make inferences about the ancestral states. For example, if the characters of interest are the nucleotides at an aligned site in DNA sequences, the Jukes–Cantor model [66] defines a transition probability for the four nucleotides at a given node in the phylogeny, conditional on the nucleotide at the ancestor of that node. The Jukes–Cantor model provides a simple example of a parametric model of nucleotide evolution—the transition probability is a parametric function of the branch length, with longer branch lengths yielding a distribution that is more nearly uniform. Given a model such as Jukes–Cantor for each branch in a phylogenetic tree, the overall joint probability of an assignment of nucleotides to all of the nodes in the tree is obtained by taking the product of the branch-wise conditional probabilities (together with a marginal probability distribution for the root). Conditioning on observed values of some of the nodes (e.g., the leaves of the tree, corresponding to extant species), classical dynamic programming algorithms (e.g., the “pruning” algorithm) can be used to infer posterior probability distributions on the states of the unobserved nodes [40].

SIFTER borrows much of the probabilistic machinery of phylogenetic analysis in the service of an inference procedure for molecular function evolution. The major new issues include the following: (1) given our choice of GO as a source of functional labels, functions are not a simple list of mutually exclusive characters, but are vertices in a DAG; (2) we require a model akin to Jukes–Cantor but appropriate for molecular function; (3) generally only a small subset of the proteins in a family are annotated, and the annotations have different degrees of reliability. We describe our approach to these issues below.

The first step of SIFTER is conventional sequence-based phylogenetic reconstruction and reconciliation.

Phylogenetic reconstruction is the computational bottleneck in the application of SIFTER. Thus, in the current implementation of SIFTER we have made use of parsimony methods instead of more computationally intense likelihood-based or Bayesian methods in phylogenetic reconstruction. This “empirical Bayes” simplification makes it possible to apply SIFTER to genome-scale problems.

In detail, the steps of phylogenetic reconstruction implemented in SIFTER are as follows. Given a query protein, we (1) find a Pfam family of a homologous domain [47], and extract the multiple sequence alignment from the Pfam database (release 12.0); (2) build a rooted phylogenetic tree with PAUP* version 4.0*b*10 [67], using parsimony with the BLOSUM50 matrix; (3) apply Forester version 1.92 [68] to estimate the location of the duplication events at the internal nodes of the phylogeny by reconciling the topological differences between a reference species tree (taken from the Pfam database) and the protein tree.

The result of this procedure is a “reconciled phylogeny,” a rooted phylogenetic tree with branch lengths and duplication events annotated at the internal nodes [45,46].

Subsequent stages of SIFTER retain these structural elements of the phylogeny, but replace the amino acid characters with vectors of molecular function annotations and place a model of molecular function evolution on the branches of the phylogeny.

We use the following process to define a vector of candidate molecular function annotations for a given query protein and for the other proteins in the phylogeny.

Given a Pfam family of a homologous domain for a query protein, we index into the GOA database [44] (we used the version of January 6, 2004) and form an initial raw list of candidate molecular functions by taking the union of the experimental annotations associated with all of the proteins in the Pfam family. We then prune this list by making use of the structure of GO in the following way. Recall that GO is organized into a DAG of functions, with the more specific function names at the leaves. Given our initial list of functions, we choose those functions that are closest to the leaves of the DAG, under the constraint that the corresponding nodes form a “nad” subset—a subset of nodes that are “not ancestors and not descendants” of each other in GO [69].

We treat the elements of this nad subset as the indices of a vector of candidate functional annotations, to be referred to as the “annotation vector” in the remainder of this section. Subsequent inferential stages of SIFTER treat this vector as a Boolean random vector. That is, we assume that each function can be asserted as either present or absent for a given protein, and we allow more than one function to be asserted as being present.

The goal is to compute posterior probabilities for all unannotated proteins in the family of interest, conditioning on experimentally derived annotations (i.e., IDA or IMP annotations) associated with some of the proteins in the family. To accommodate the fact that IDA annotations may be more reliable than IMP annotations according to the experiments by which they are generated, and to allow users to make use of other, possibly less reliable, annotations, SIFTER distinguishes between a notion of “true function” and “annotated function,” and defines a likelihood function linking these variables. In particular, the current implementation of SIFTER defines expert-elicited probabilities that an experimentally derived annotation is correct given the method of annotation: IDA annotations are treated as having a likelihood of 0.9 of being correct, and IMP as having a likelihood of 0.8.

GOA database annotations are not restricted to the leaves of the ontology but can be found throughout the DAG. To incorporate all such annotations in SIFTER, we need to propagate annotations to the nad subset. In particular, annotations at nodes that are ancestors to nad nodes need to be propagated downward to the nad nodes. (By definition, there can be no annotated descendants of nad nodes.) We do this by treating evidence at an ancestor node as evidence for all possible combinations of its descendants, according to the distribution *Q*(*S*) = 1/η^|*S*|^, where *S* is an arbitrary subset of the nad nodes, |*S*| is the cardinality of *S,* and the value of η is fixed by the requirement that 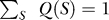 . Finally, to combine annotations at a given node we take one minus the product of their errors (where error is one minus their likelihood).

**Figure 6:**
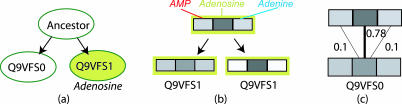
A Depiction of a Fragment of a Phylogeny and the Noisy-OR Model (A) Two proteins, Q9VFS0 and Q9VFS1, both from *Drosophila melanogaster,* related by a common ancestor protein. (B) Protein Q9VFS1 has a functional observation for adenosine deaminase (the center rectangle). Also shown are the posterior probabilities for each molecular function as grayscale (white indicating zero and black indicating one) of the annotation vector after inference. Each component of the vector corresponds to a particular deaminase substrate. (C) The noisy-OR model that underlies the inference procedure. We focus on the adenosine deaminase random variable in protein Q9VFS0. The transition probability for this random variable depends on all of the ancestor random variables and the transition parameters *q_m,n_*.

We turn to a description of the model of molecular function evolution that SIFTER associates with the branches of the phylogeny. For each node in the phylogeny, corresponding to a single protein, this model defines the conditional probability for the vector of function annotations at the node, conditioning on the value of the vector of function annotations at the ancestor of the node. Figure 6 provides an overview of the model and its role in the inference procedure.

We chose a statistical model known as a loglinear model for the model of function evolution. We make no claims for any theoretical justification of this model. It is simply a phenomenological model that captures in broad outlines some of the desiderata of an evolutionary model for function and has worked well in practice in our phylogenomic setting.

Let *X_i_* denote the Boolean vector of candidate molecular function annotations for node *i* and let 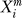 denote the *m*th component of this vector. Let *M* denote the number of components of this vector. Let π*_i_* denote the immediate ancestor of node *i* in the phylogeny, so that 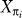 denotes the annotation vector at the ancestor. We define the transition probability associated with the branch from π*_i_* to *i* as follows:


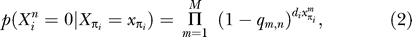


where *d_i_* and *q_m,n_* are parametric functions of branch lengths in the phylogeny and path lengths in GO, respectively. This functional form is known as a “noisy-OR” function [70], and it has the following interpretation. Suppose that 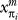 is equal to one for only a single value of *m* and is equal to zero for all other values of *m*. (Thus, a single function is asserted as present for the parent.) Suppose that *d_i_* is equal to one. Then the probability that node *i* has the *n*th function (i.e., that 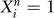 ) is equal to *q_m,n_*. Thus, *q_m,n_* has an interpretation as a local transition probability between the *m*th function and the *n*th function. The multiplication in Equation 2 corresponds to an assumption of independence (specifically, independence of the events that an ancestor function *m* fails to trigger a function *n* in the descendant).

To capture the notion that a transition should be less probable the less “similar” two functions are, we defined *q_m,n_* to be a decreasing function of the path length *l_m,n_* in GO. Specifically, we let 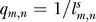 *,* where *s* is a free parameter. This parameter is taken to be different for speciation and duplication events; in particular, it is larger in the latter case, corresponding to the phylogenomic assumption that evolutionary transitions are more rapid following a duplication event. To set the parameters *s*_speciation_ and *s*_duplication_, we can in principle make use of resampling methods such as cross-validation or the bootstrap. In the case of the deaminase family, however, the number of observed data points (five) is too small for these methods to yield reasonable results, and in our analyses of this family we simply fixed the parameters to the values *s*_speciation_ = 3 and *s*_duplication_ = 4 and did not consider other values. For the 100-family dataset, we ran each family with a few different parameter settings, because the number of annotations available for the families was in general prohibitively small, and fixed them at the set of values that produced predictions most closely aligned with the non-experimental annotations from the GOA database. We define *q_m,m_* = (1/r)*^s^* ^/2^ for self-transitions; this normalizes the self-transition probability with respect to the number of components of the annotation vector.

We also need to parameterize the transition rate as a function of the branch length in the phylogeny. This is achieved by defining *d_i_* to be a decreasing nonlinear function of the branch length. (Thus, for greater branch lengths, transitions become more probable.) Specifically, we set 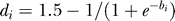 , where *b_i_* is the most parsimonious number of amino acid mutations along the branch from π*_i_* to *i*.

Having defined a probabilistic transition model for the branches of the phylogeny, and having defined a mechanism whereby evidence is incorporated into the tree, it remains to solve the problem of computing the posterior probability of the unobserved functions in the tree conditional on the evidence.

This problem is readily solved using standard probabilistic propagation algorithms. Specifically, all posterior probabilities can be obtained in linear time via the classical pruning algorithm [40], also known as (a special case of) the junction tree algorithm [37]. This algorithm propagates probabilistic “messages” from the leaves of the tree to the root, and from the root back to the leaves, performing a constant number of operations at each node. The computational complexity of the algorithm is thus linear in the number of leaves in the tree.

### Methods for comparison.

The BLAST version 2.2.4 [4] assessment was performed on the non-redundant set of proteins from Swiss-Prot downloaded from the NCBI Web site on March 7, 2004. We ran BLASTP with an *E*-value cutoff of 0.01. We transferred annotation from the highest scoring non-identity protein (BLASTB), which was determined by checking the alignment for 100% identity and identical species name. We also transferred annotation from the highest scoring annotated non-identity protein (BLASTA), which was the highest scoring non-identity protein that had a functional description (i.e., not “hypothetical protein” or “unknown function”). Phrases modifying a functional annotation such as “putative” and “*-*related” were ignored. An annotation including an EC number was considered unambiguous.

To build the ROC plots for the BLASTC comparison, for each protein in the selected families we searched the BLAST output for the highest scoring sequence (with the most significant *E*-value) that had a function description from the appropriate set: for the deaminase family we searched for “adenosine deaminase,” “adenine deaminase,” “AMP deaminase,” and, for the results on multiple functions, “growth factor activity.” A reference could also be in the form of an EC number or unambiguous phrase (e.g., “growth and transcription activator” was interpreted as “growth factor activity”). We plotted the false positives (one minus specificity) versus true positives (sensitivity) as the acceptance cutoff for *E*-values ranges from 0.01 to zero, where proteins were annotated with a function if the most significant *E*-value for a protein with that particular function was less than the acceptance cutoff.

To build the BLASTC set of annotations for the set of 18,736 proteins from the 100-family dataset, we built a keyword search with 260 GO terms, including all of the terms from the SIFTER analysis and other terms common to the BLAST search results. From this keyword search we extracted a set of terms ranked by *E*-values, facilitated by BioPerl [71]. The first of the ranked set of terms was then compared against predictions from alternate methods, using GO molecular function term comparisons. The full set of data including the keyword search code is available in Dataset S1.

For GeneQuiz, we ran each member of the AMP/adenosine deaminase and lactate/malate dehydrogenase families on the GeneQuiz server (publicly available at EBI) from August 22, 2004, to September 1, 2004 [13]. The function predictions from GeneQuiz are not based on an ontology, and we manually converted them to equivalent GO numbers. If there was an EC number, the annotation correlated exactly with a GO term. We ignored phrases such as “putative,” “fragment,” or “weakly similar to,” and only interpreted the functional words.

For GOtcha, we ran the first publicly available version of the GOtcha software [6], kindly provided by D. Martin, on the set of 18,736 proteins from the 100-family dataset. We searched the protein sequences against all seven available genome databases, gathering results using all annotations (GOtcha), excluding IEA annotations (GOtcha-ni), and finally including only IMP and IDA annotations (GOtcha-exp). The output is a ranked list of GO terms. It is a property of GOtcha that the top-ranked terms are the more general ones (e.g., “molecular function,” the most general term in the molecular function ontology, is always ranked first). We parsed out the molecular function annotations, retaining the relative rank of each term, and discarding terms that were too general but had compatible terms ranked below them. We broke ties in rank in favor of the correct term (for the deaminase family). We compared the top member of this ranked list against predictions from the other methods.

For Orthostrapper [19], we ran the version from February 6, 2002, on each of the 100 Pfam families in our dataset. Species 1 and species 2 were all of the proteins with any type of GO annotation containing the proteins from eukaryotes and non-eukaryotes, respectively, when both sets were not empty (and mammals and non-mammals otherwise). We clustered the bootstrapped analysis according to the cluster program in Orthostrapper, using a bootstrap cutoff of 750 and then using a cutoff of one, resulting in the statistically significant clusters and non-statistically significant clusters, respectively.

In each cluster, we transferred all experimentally derived GO annotations from member proteins onto the remaining proteins without experimentally derived GO annotations. If a cluster did not contain a protein with an experimentally derived GO annotation, no functions were transferred; if a protein was present in multiple clusters, it would receive annotations transferred within each of those clusters. This method yields an unranked set of predictions for each protein.

### Deaminase activity assays.

Purified Q8IJA9_PLAFA was the kind gift of Erica Boni, Chris Mehlin, and Wim Hol of the Stuctural Genomics of Pathogenic Protozoa project at the University of Washington. Adenosine and adenine were from Sigma-Aldrich (St. Louis, Missouri, United States), AMP was from Schwarz Laboratories (Mt. Vernon, New York, United States), and monobasic and dibasic potassium phosphate were from EMD Chemicals (Gibbstown, New Jersey, United States).

The loss of absorbance at 265 nm was monitored with an Agilent Technologies (Palo Alto, California, United States) 8453 spectrophotometer. The Δɛ between substrate adenosine and product inosine is 7,740 AU M^−1^ cm^−1^ [72].

## Supporting Information

Dataset S1 SIFTER Supplemental Data (7.5 MB TGZ)

### Accession Numbers

The Swiss-Prot (http://www.ebi.ac.uk/swissprot/) accession number for *H. sapiens* adenosine deaminase is P00813 and for *P. falciparum* adenosine deaminase is Q8IJA9. The Pfam (http://www.sanger.ac.uk/Software/Pfam/) accession number for the AMP/adenosine deaminase family is PF00962.

## Abbreviations

AMP: adenosine-5′-monophosphate
DAG: directed acyclic graph
EC: Enzyme Commission
GO: Gene Ontology
GOA: Gene Ontology annotation
LDH: lactate dehydrogenase
MDH: malate dehydrogenase
ROC: receiver operating characteristic
