# Supplementary material for: Protein Molecular Function Prediction by Bayesian Phylogenomics
Source: PLoS Comput Biol. 2005 Oct 7;1(5):e45. doi: 10.1371/journal.pcbi.0010045 (PMC1246806; doi:10.1371/journal.pcbi.0010045)
Supplement: Supplementary file 1 [file pcbi.0010045.sd001.tgz › sifter.html]

Statistical Phylogenomics (SIFTER) Supplemental Data


# Statistical Phylogenomics (SIFTER) Supplemental Data

---

### SIFTER: Statistical Inference of Function Through Evolutionary Relationships

  
**Background:** The post-genomic era has revealed sequences for
many proteins, but the rate of sequence acquisition far surpasses the
rate of accurate protein function determination. Computational methods
for protein annotation are applied to fill the gap between sequence
availability and experimental functional
characterization. Unfortunately, many current methods used for
function annotation rely on sequence comparison methods, which obscure
evolutionary history and systematically produce incorrect predictions.
Phylogenomics, which leverages knowledge about how protein function
evolves, is widely-recognized to be the current best solution to
protein function annotation, although because it is manual, it cannot
be applied universally.   
**Methodology/Principal Findings:** We present a statistical
graphical model of molecular function evolution to infer specific
function for protein sequences. Based on phylogenomic principles,
SIFTER (Statistical Inference of Function Through Evolutionary
Relationships) accurately predicts function for protein family members
using a phylogeny and available functional annotations. We performed
SIFTER analyses on 100 Pfam families to assess its performance across
a diverse set of protein families. We compare the predictions from
this diverse set of proteins to predictions from BLAST, GOtcha, and
orthostrapper, and the Gene Ontology annotation database. We selected
the AMP/adenosine deaminase family family to explore the predictions
in detail, including comparing the predictions against published
experimental functional characterizations. Based on function
descriptions for 3% of the proteins in this family, SIFTER achieves
96% accuracy in predicting function on this gold standard dataset. We
studied the robustness of SIFTER under various alignment and phylogeny
reconstruction methods and found minimal variance in the outcome. We
performed a kinetic analysis to confirm the SIFTER prediction of
deaminase activity with the substrate adenosine (and absence of
activity with substrates AMP and adenine) for a protein in the
deaminase family from *Plasmodium falciparum*.   
**Conclusions/Significance**: The accuracy of function predictions from
SIFTER is a significant improvement over methods such as BLAST
(32% - 75%) and GeneQuiz (64% - 68%). Output from SIFTER,
including a statistical measure of certainty for each prediction,
enables biologists to explore the evolutionary history of a protein
family. The results illustrate the predictive power of exploiting a
statistical model of function evolution in phylogenomic problems.
  
[PDF]

---

**Plot of SIFTER error versus BLASTA, BLASTB and GeneQuiz for four different prediction problems.**


---

Deaminase Family
  
A set of data related to the in-depth exploration of the Pfam AMP/adenosine deaminase family (PF00962). This set includes the BLAST and GeneQuiz comparison results summaries and the set of references in PubMed that were used for the held out annotation data set. Posterior probabilities, parameter sets, and predictions from SIFTER are included below in the "Phylogeny data and results" section for this family. This section also includes the raw kinetics data for the experimental characterization of the protein Q9IJA8\_PLAFA.

---

Phylogeny data and results
  
We have included a list of all of the families analyzed using SIFTER, including their gene trees (built using PAUP\*), species trees (from Pfam, except where noted), reconciled trees (built using Forester), results (posterior probabilities for each node/protein, GOA annotations, orthostrapper predictions, GOtcha predictions, and BLAST predictions), and inference parameters.
  


---

Barbara Engelhardt


Last modified: Mon Dec 6 10:18:35 PST 2004
