# Supplementary material for: Protein Molecular Function Prediction by Bayesian Phylogenomics
Source: PLoS Comput Biol. 2005 Oct 7;1(5):e45. doi: 10.1371/journal.pcbi.0010045 (PMC1246806; doi:10.1371/journal.pcbi.0010045)
Supplement: Supplementary file 1 [file pcbi.0010045.sd001.tgz › sifter/100trees.html~]

Additional Phylogenies


# Additional Phylogenies

Gene trees, species trees, and reconciled trees can all be viewed with A Tree Viewer (ATV).
  
The column order of the GO functions for the posterior probabilities is given in either parameters document.
  
The set of proteins to index for each family in the complete set of GOtcha runs are available for each family below.
The complete set of GOtcha runs: [tar];
the complete set of GOtcha No IEA runs: [tar];
  
The format of the orthostrapper file is: protein\_name \* GOA annotations \* non-significant annotations\* significant annotations. A "-1" means that no annotation was transferred.
  
The keyword search for BLASTC runs: [perl file];
  


- PF00013: KH domain   
  [gene tree]
  [species tree]
  [reconciled tree]
  [posterior probabilities]
  [speciation parameters]
  [duplication parameters]
  [orthostrapper/GOA]
  [BLAST]
  [GOtcha proteins]
  [GOtcha]
  [GOtcha No IEA]- PF00048: Small cytokines (intecrine/chemokine), interleukin-8 like   
    [gene tree]
    [species tree]
    [reconciled tree]
    [posterior probabilities]
    [speciation parameters]
    [duplication parameters]
    [orthostrapper/GOA]
    [BLAST]
    [GOtcha proteins]
    [GOtcha]
    [GOtcha No IEA]- PF00052: Laminin B (Domain IV)   
      [gene tree]
      [species tree]
      [reconciled tree]
      [posterior probabilities]
      [speciation parameters]
      [duplication parameters]
      [orthostrapper/GOA]
      [BLAST]
      [GOtcha proteins]
      [GOtcha]
      [GOtcha No IEA]- PF00053: Laminin EGF-like (Domains III and V)   
        [gene tree]
        [species tree]
        [reconciled tree]
        [posterior probabilities]
        [speciation parameters]
        [duplication parameters]
        [orthostrapper/GOA]
        [BLAST]
        [GOtcha proteins]
        [GOtcha]
        [GOtcha No IEA]- PF00055: Laminin N-terminal (Domain VI)   
          [gene tree]
          [species tree]
          [reconciled tree]
          [posterior probabilities]
          [speciation parameters]
          [duplication parameters]
          [orthostrapper/GOA]
          [BLAST]
          [GOtcha proteins]
          [GOtcha]
          [GOtcha No IEA]- PF00056: lactate/malate dehydrogenase, NAD binding domain   
            [gene tree]
            [species tree]
            [reconciled tree]
            [posterior probabilities]
            [speciation parameters]
            [duplication parameters]
            [orthostrapper/GOA]
            [BLAST]
            [GOtcha proteins]
            [GOtcha]
            [GOtcha No IEA]- PF00057: Low-density lipoprotein receptor domain class A   
              [gene tree]
              [species tree]
              [reconciled tree]
              [posterior probabilities]
              [speciation parameters]
              [duplication parameters]
              [orthostrapper/GOA]
              [BLAST]
              [GOtcha proteins]
              [GOtcha]
              [GOtcha No IEA]- PF00060: Ligand-gated ion channel   
                [gene tree]
                [species tree]
                [reconciled tree]
                [posterior probabilities]
                [speciation parameters]
                [duplication parameters]
                [orthostrapper/GOA]
                [BLAST]
                [GOtcha proteins]
                [GOtcha]
                [GOtcha No IEA]- PF00102: Protein-tyrosine phosphatase   
                  [gene tree]
                  [species tree]
                  [reconciled tree]
                  [posterior probabilities]
                  [speciation parameters]
                  [duplication parameters]
                  [orthostrapper/GOA]
                  [BLAST]
                  [GOtcha proteins]
                  [GOtcha]
                  [GOtcha No IEA]- PF00104: Ligand-binding domain of nuclear hormone receptor   
                    [gene tree]
                    [species tree]
                    [reconciled tree]
                    [posterior probabilities]
                    [speciation parameters]
                    [duplication parameters]
                    [orthostrapper/GOA]
                    [BLAST]
                    [GOtcha proteins]
                    [GOtcha]
                    [GOtcha No IEA]- PF00130: Phorbol esters/diacylglycerol binding domain (C1 domain)   
                      [gene tree]
                      [species tree]
                      [reconciled tree]
                      [posterior probabilities]
                      [speciation parameters]
                      [duplication parameters]
                      [orthostrapper/GOA]
                      [BLAST]
                      [GOtcha proteins]
                      [GOtcha]
                      [GOtcha No IEA]- PF00134: Cyclin, N-terminal domain   
                        [gene tree]
                        [species tree]
                        [reconciled tree]
                        [posterior probabilities]
                        [speciation parameters]
                        [duplication parameters]
                        [orthostrapper/GOA]
                        [BLAST]
                        [GOtcha proteins]
                        [GOtcha]
                        [GOtcha No IEA]- PF00160: Cyclophilin type peptidyl-prolyl cis-trans isomerase   
                          [gene tree]
                          [species tree]
                          [reconciled tree]
                          [posterior probabilities]
                          [speciation parameters]
                          [duplication parameters]
                          [orthostrapper/GOA]
                          [BLAST]
                          [GOtcha proteins]
                          [GOtcha]
                          [GOtcha No IEA]- PF00172: Fungal Zn(2)-Cys(6) binuclear cluster domain   
                            [gene tree]
                            [species tree]
                            [reconciled tree]
                            [posterior probabilities]
                            [speciation parameters]
                            [duplication parameters]
                            [orthostrapper/GOA]
                            [BLAST]
                            [GOtcha proteins]
                            [GOtcha]
                            [GOtcha No IEA]- PF00211: Adenylate and Guanylate cyclase catalytic domain   
                              [gene tree]
                              [species tree]
                              [reconciled tree]
                              [posterior probabilities]
                              [speciation parameters]
                              [duplication parameters]
                              [orthostrapper/GOA]
                              [BLAST]
                              [GOtcha proteins]
                              [GOtcha]
                              [GOtcha No IEA]- PF00225: Kinesin motor domain   
                                [gene tree]
                                [species tree]
                                [reconciled tree]
                                [posterior probabilities]
                                [speciation parameters]
                                [duplication parameters]
                                [orthostrapper/GOA]
                                [BLAST]
                                [GOtcha proteins]
                                [GOtcha]
                                [GOtcha No IEA]- PF00241: Cofilin/tropomyosin-type actin-binding protein   
                                  [gene tree]
                                  [species tree]
                                  [reconciled tree]
                                  [posterior probabilities]
                                  [speciation parameters]
                                  [duplication parameters]
                                  [orthostrapper/GOA]
                                  [BLAST]
                                  [GOtcha proteins]
                                  [GOtcha]
                                  [GOtcha No IEA]- PF00250: Fork head domain   
                                    [gene tree]
                                    [species tree]
                                    [reconciled tree]
                                    [posterior probabilities]
                                    [speciation parameters]
                                    [duplication parameters]
                                    [orthostrapper/GOA]
                                    [BLAST]
                                    [GOtcha proteins]
                                    [GOtcha]
                                    [GOtcha No IEA]- PF00289: gen   
                                      [gene tree]
                                      [species tree]
                                      [reconciled tree]
                                      [posterior probabilities]
                                      [speciation parameters]
                                      [duplication parameters]
                                      [orthostrapper/GOA]
                                      [BLAST]
                                      [GOtcha proteins]
                                      [GOtcha]
                                      [GOtcha No IEA]- PF00307: Calponin homology (CH) domain   
                                        [gene tree]
                                        [species tree]
                                        [reconciled tree]
                                        [posterior probabilities]
                                        [speciation parameters]
                                        [duplication parameters]
                                        [orthostrapper/GOA]
                                        [BLAST]
                                        [GOtcha proteins]
                                        [GOtcha]
                                        [GOtcha No IEA]- PF00313: 'Cold-shock' DNA-binding domain   
                                          [gene tree]
                                          [species tree]
                                          [reconciled tree]
                                          [posterior probabilities]
                                          [speciation parameters]
                                          [duplication parameters]
                                          [orthostrapper/GOA]
                                          [BLAST]
                                          [GOtcha proteins]
                                          [GOtcha]
                                          [GOtcha No IEA]- PF00320: GATA zinc finger   
                                            [gene tree]
                                            [species tree]
                                            [reconciled tree]
                                            [posterior probabilities]
                                            [speciation parameters]
                                            [duplication parameters]
                                            [orthostrapper/GOA]
                                            [BLAST]
                                            [GOtcha proteins]
                                            [GOtcha]
                                            [GOtcha No IEA]- PF00339: Arrestin (or S-antigen), N-terminal domain   
                                              [gene tree]
                                              [species tree]
                                              [reconciled tree]
                                              [posterior probabilities]
                                              [speciation parameters]
                                              [duplication parameters]
                                              [orthostrapper/GOA]
                                              [BLAST]
                                              [GOtcha proteins]
                                              [GOtcha]
                                              [GOtcha No IEA]- PF00349: Hexokinase   
                                                [gene tree]
                                                [species tree]
                                                [reconciled tree]
                                                [posterior probabilities]
                                                [speciation parameters]
                                                [duplication parameters]
                                                [orthostrapper/GOA]
                                                [BLAST]
                                                [GOtcha proteins]
                                                [GOtcha]
                                                [GOtcha No IEA]- PF00357: Sodium:dicarboxylate symporter family   
                                                  [gene tree]
                                                  [species tree]
                                                  [reconciled tree]
                                                  [posterior probabilities]
                                                  [speciation parameters]
                                                  [duplication parameters]
                                                  [orthostrapper/GOA]
                                                  [BLAST]
                                                  [GOtcha proteins]
                                                  [GOtcha]
                                                  [GOtcha No IEA]- PF00397: WW domain   
                                                    [gene tree]
                                                    [species tree]
                                                    [reconciled tree]
                                                    [posterior probabilities]
                                                    [speciation parameters]
                                                    [duplication parameters]
                                                    [orthostrapper/GOA]
                                                    [BLAST]
                                                    [GOtcha proteins]
                                                    [GOtcha]
                                                    [GOtcha No IEA]- PF00408: Phosphoglucomutase/phosphomannomutase, C-terminal domain   
                                                      [gene tree]
                                                      [species tree]
                                                      [reconciled tree]
                                                      [posterior probabilities]
                                                      [speciation parameters]
                                                      [duplication parameters]
                                                      [orthostrapper/GOA]
                                                      [BLAST]
                                                      [GOtcha proteins]
                                                      [GOtcha]
                                                      [GOtcha No IEA]- PF00433: Protein kinase C terminal domain   
                                                        [gene tree]
                                                        [species tree]
                                                        [reconciled tree]
                                                        [posterior probabilities]
                                                        [speciation parameters]
                                                        [duplication parameters]
                                                        [orthostrapper/GOA]
                                                        [BLAST]
                                                        [GOtcha proteins]
                                                        [GOtcha]
                                                        [GOtcha No IEA]- PF00435: Spectrin repeat   
                                                          [gene tree]
                                                          [species tree]
                                                          [reconciled tree]
                                                          [posterior probabilities]
                                                          [speciation parameters]
                                                          [duplication parameters]
                                                          [orthostrapper/GOA]
                                                          [BLAST]
                                                          [GOtcha proteins]
                                                          [GOtcha]
                                                          [GOtcha No IEA]- PF00447: HSF-type DNA-binding   
                                                            [gene tree]
                                                            [species tree]
                                                            [reconciled tree]
                                                            [posterior probabilities]
                                                            [speciation parameters]
                                                            [duplication parameters]
                                                            [orthostrapper/GOA]
                                                            [BLAST]
                                                            [GOtcha proteins]
                                                            [GOtcha]
                                                            [GOtcha No IEA]- PF00454: Phosphatidylinositol 3- and 4-kinase   
                                                              [gene tree]
                                                              [species tree]
                                                              [reconciled tree]
                                                              [posterior probabilities]
                                                              [speciation parameters]
                                                              [duplication parameters]
                                                              [orthostrapper/GOA]
                                                              [BLAST]
                                                              [GOtcha proteins]
                                                              [GOtcha]
                                                              [GOtcha No IEA]- PF00498: FHA domain   
                                                                [gene tree]
                                                                [species tree]
                                                                [reconciled tree]
                                                                [posterior probabilities]
                                                                [speciation parameters]
                                                                [duplication parameters]
                                                                [orthostrapper/GOA]
                                                                [BLAST]
                                                                [GOtcha proteins]
                                                                [GOtcha]
                                                                [GOtcha No IEA]- PF00514: Armadillo/beta-catenin-like repeat   
                                                                  [gene tree]
                                                                  [species tree]
                                                                  [reconciled tree]
                                                                  [posterior probabilities]
                                                                  [speciation parameters]
                                                                  [duplication parameters]
                                                                  [orthostrapper/GOA]
                                                                  [BLAST]
                                                                  [GOtcha proteins]
                                                                  [GOtcha]
                                                                  [GOtcha No IEA]- PF00533: BRCA1 C Terminus (BRCT) domain   
                                                                    [gene tree]
                                                                    [species tree]
                                                                    [reconciled tree]
                                                                    [posterior probabilities]
                                                                    [speciation parameters]
                                                                    [duplication parameters]
                                                                    [orthostrapper/GOA]
                                                                    [BLAST]
                                                                    [GOtcha proteins]
                                                                    [GOtcha]
                                                                    [GOtcha No IEA]- PF00536: SAM domain (Sterile alpha motif)   
                                                                      [gene tree]
                                                                      [species tree]
                                                                      [reconciled tree]
                                                                      [posterior probabilities]
                                                                      [speciation parameters]
                                                                      [duplication parameters]
                                                                      [orthostrapper/GOA]
                                                                      [BLAST]
                                                                      [GOtcha proteins]
                                                                      [GOtcha]
                                                                      [GOtcha No IEA]- PF00554: Rel homology domain (RHD)   
                                                                        [gene tree]
                                                                        [species tree]
                                                                        [reconciled tree]
                                                                        [posterior probabilities]
                                                                        [speciation parameters]
                                                                        [duplication parameters]
                                                                        [orthostrapper/GOA]
                                                                        [BLAST]
                                                                        [GOtcha proteins]
                                                                        [GOtcha]
                                                                        [GOtcha No IEA]- PF00568: WH1 domain   
                                                                          [gene tree]
                                                                          [species tree]
                                                                          [reconciled tree]
                                                                          [posterior probabilities]
                                                                          [speciation parameters]
                                                                          [duplication parameters]
                                                                          [orthostrapper/GOA]
                                                                          [BLAST]
                                                                          [GOtcha proteins]
                                                                          [GOtcha]
                                                                          [GOtcha No IEA]- PF00609: Diacylglycerol kinase accessory domain   
                                                                            [gene tree]
                                                                            [species tree]
                                                                            [reconciled tree]
                                                                            [posterior probabilities]
                                                                            [speciation parameters]
                                                                            [duplication parameters]
                                                                            [orthostrapper/GOA]
                                                                            [BLAST]
                                                                            [GOtcha proteins]
                                                                            [GOtcha]
                                                                            [GOtcha No IEA]- PF00611: Fes/CIP4 homology domain   
                                                                              [gene tree]
                                                                              [species tree]
                                                                              [reconciled tree]
                                                                              [posterior probabilities]
                                                                              [speciation parameters]
                                                                              [duplication parameters]
                                                                              [orthostrapper/GOA]
                                                                              [BLAST]
                                                                              [GOtcha proteins]
                                                                              [GOtcha]
                                                                              [GOtcha No IEA]- PF00618: Guanine nucleotide exchange factor for Ras-like GTPases; N-terminal motif   
                                                                                [gene tree]
                                                                                [species tree]
                                                                                [reconciled tree]
                                                                                [posterior probabilities]
                                                                                [speciation parameters]
                                                                                [duplication parameters]
                                                                                [orthostrapper/GOA]
                                                                                [BLAST]
                                                                                [GOtcha proteins]
                                                                                [GOtcha]
                                                                                [GOtcha No IEA]- PF00620: RhoGAP domain   
                                                                                  [gene tree]
                                                                                  [species tree]
                                                                                  [reconciled tree]
                                                                                  [posterior probabilities]
                                                                                  [speciation parameters]
                                                                                  [duplication parameters]
                                                                                  [orthostrapper/GOA]
                                                                                  [BLAST]
                                                                                  [GOtcha proteins]
                                                                                  [GOtcha]
                                                                                  [GOtcha No IEA]- PF00621: RhoGEF domain   
                                                                                    [gene tree]
                                                                                    [species tree]
                                                                                    [reconciled tree]
                                                                                    [posterior probabilities]
                                                                                    [speciation parameters]
                                                                                    [duplication parameters]
                                                                                    [orthostrapper/GOA]
                                                                                    [BLAST]
                                                                                    [GOtcha proteins]
                                                                                    [GOtcha]
                                                                                    [GOtcha No IEA]- PF00632: HECT-domain (ubiquitin-transferase)   
                                                                                      [gene tree]
                                                                                      [species tree]
                                                                                      [reconciled tree]
                                                                                      [posterior probabilities]
                                                                                      [speciation parameters]
                                                                                      [duplication parameters]
                                                                                      [orthostrapper/GOA]
                                                                                      [BLAST]
                                                                                      [GOtcha proteins]
                                                                                      [GOtcha]
                                                                                      [GOtcha No IEA]- PF00652: QXW lectin repeat   
                                                                                        [gene tree]
                                                                                        [species tree]
                                                                                        [reconciled tree]
                                                                                        [posterior probabilities]
                                                                                        [speciation parameters]
                                                                                        [duplication parameters]
                                                                                        [orthostrapper/GOA]
                                                                                        [BLAST]
                                                                                        [GOtcha proteins]
                                                                                        [GOtcha]
                                                                                        [GOtcha No IEA]- PF00656: Caspase domain   
                                                                                          [gene tree]
                                                                                          [species tree]
                                                                                          [reconciled tree]
                                                                                          [posterior probabilities]
                                                                                          [speciation parameters]
                                                                                          [duplication parameters]
                                                                                          [orthostrapper/GOA]
                                                                                          [BLAST]
                                                                                          [GOtcha proteins]
                                                                                          [GOtcha]
                                                                                          [GOtcha No IEA]- PF00685: Sulfotransferase domain   
                                                                                            [gene tree]
                                                                                            [species tree]
                                                                                            [reconciled tree]
                                                                                            [posterior probabilities]
                                                                                            [speciation parameters]
                                                                                            [duplication parameters]
                                                                                            [orthostrapper/GOA]
                                                                                            [BLAST]
                                                                                            [GOtcha proteins]
                                                                                            [GOtcha]
                                                                                            [GOtcha No IEA]- PF00777: Glycosyltransferase family 29 (sialyltransferase)   
                                                                                              [gene tree]
                                                                                              [species tree]
                                                                                              [reconciled tree]
                                                                                              [posterior probabilities]
                                                                                              [speciation parameters]
                                                                                              [duplication parameters]
                                                                                              [orthostrapper/GOA]
                                                                                              [BLAST]
                                                                                              [GOtcha proteins]
                                                                                              [GOtcha]
                                                                                              [GOtcha No IEA]- PF00780: CNH domain   
                                                                                                [gene tree]
                                                                                                [species tree]
                                                                                                [reconciled tree]
                                                                                                [posterior probabilities]
                                                                                                [speciation parameters]
                                                                                                [duplication parameters]
                                                                                                [orthostrapper/GOA]
                                                                                                [BLAST]
                                                                                                [GOtcha proteins]
                                                                                                [GOtcha]
                                                                                                [GOtcha No IEA]- PF00782: Dual specificity phosphatase, catalytic domain   
                                                                                                  [gene tree]
                                                                                                  [species tree]
                                                                                                  [reconciled tree]
                                                                                                  [posterior probabilities]
                                                                                                  [speciation parameters]
                                                                                                  [duplication parameters]
                                                                                                  [orthostrapper/GOA]
                                                                                                  [BLAST]
                                                                                                  [GOtcha proteins]
                                                                                                  [GOtcha]
                                                                                                  [GOtcha No IEA]- PF00786: P21-Rho-binding domain   
                                                                                                    [gene tree]
                                                                                                    [species tree]
                                                                                                    [reconciled tree]
                                                                                                    [posterior probabilities]
                                                                                                    [speciation parameters]
                                                                                                    [duplication parameters]
                                                                                                    [orthostrapper/GOA]
                                                                                                    [BLAST]
                                                                                                    [GOtcha proteins]
                                                                                                    [GOtcha]
                                                                                                    [GOtcha No IEA]- PF00788: Ras association (RalGDS/AF-6) domain   
                                                                                                      [gene tree]
                                                                                                      [species tree]
                                                                                                      [reconciled tree]
                                                                                                      [posterior probabilities]
                                                                                                      [speciation parameters]
                                                                                                      [duplication parameters]
                                                                                                      [orthostrapper/GOA]
                                                                                                      [BLAST]
                                                                                                      [GOtcha proteins]
                                                                                                      [GOtcha]
                                                                                                      [GOtcha No IEA]- PF00850: Histone deacetylase domain   
                                                                                                        [gene tree]
                                                                                                        [species tree]
                                                                                                        [reconciled tree]
                                                                                                        [posterior probabilities]
                                                                                                        [speciation parameters]
                                                                                                        [duplication parameters]
                                                                                                        [orthostrapper/GOA]
                                                                                                        [BLAST]
                                                                                                        [GOtcha proteins]
                                                                                                        [GOtcha]
                                                                                                        [GOtcha No IEA]- PF00856: SET domain   
                                                                                                          [gene tree]
                                                                                                          [species tree]
                                                                                                          [reconciled tree]
                                                                                                          [posterior probabilities]
                                                                                                          [speciation parameters]
                                                                                                          [duplication parameters]
                                                                                                          [orthostrapper/GOA]
                                                                                                          [BLAST]
                                                                                                          [GOtcha proteins]
                                                                                                          [GOtcha]
                                                                                                          [GOtcha No IEA]- PF00916: Sulfate transporter family   
                                                                                                            [gene tree]
                                                                                                            [species tree]
                                                                                                            [reconciled tree]
                                                                                                            [posterior probabilities]
                                                                                                            [speciation parameters]
                                                                                                            [duplication parameters]
                                                                                                            [orthostrapper/GOA]
                                                                                                            [BLAST]
                                                                                                            [GOtcha proteins]
                                                                                                            [GOtcha]
                                                                                                            [GOtcha No IEA]- PF00929: Exonuclease   
                                                                                                              [gene tree]
                                                                                                              [species tree]
                                                                                                              [reconciled tree]
                                                                                                              [posterior probabilities]
                                                                                                              [speciation parameters]
                                                                                                              [duplication parameters]
                                                                                                              [orthostrapper/GOA]
                                                                                                              [BLAST]
                                                                                                              [GOtcha proteins]
                                                                                                              [GOtcha]
                                                                                                              [GOtcha No IEA]- PF00962: Adenosine/AMP deaminase   
                                                                                                                [gene tree]
                                                                                                                [species tree]
                                                                                                                [reconciled tree]
                                                                                                                [posterior probabilities]
                                                                                                                [speciation parameters]
                                                                                                                [duplication parameters]
                                                                                                                [orthostrapper/GOA]
                                                                                                                [BLAST]
                                                                                                                [GOtcha proteins]
                                                                                                                [GOtcha]
                                                                                                                [GOtcha No IEA]- PF01094: Receptor family ligand binding region   
                                                                                                                  [gene tree]
                                                                                                                  [species tree]
                                                                                                                  [reconciled tree]
                                                                                                                  [posterior probabilities]
                                                                                                                  [speciation parameters]
                                                                                                                  [duplication parameters]
                                                                                                                  [orthostrapper/GOA]
                                                                                                                  [BLAST]
                                                                                                                  [GOtcha proteins]
                                                                                                                  [GOtcha]
                                                                                                                  [GOtcha No IEA]- PF01150: GDA1/CD39 (nucleoside phosphatase) family   
                                                                                                                    [gene tree]
                                                                                                                    [species tree]
                                                                                                                    [reconciled tree]
                                                                                                                    [posterior probabilities]
                                                                                                                    [speciation parameters]
                                                                                                                    [duplication parameters]
                                                                                                                    [orthostrapper/GOA]
                                                                                                                    [BLAST]
                                                                                                                    [GOtcha proteins]
                                                                                                                    [GOtcha]
                                                                                                                    [GOtcha No IEA]- PF01214: Casein kinase II regulatory subunit   
                                                                                                                      [gene tree]
                                                                                                                      [species tree]
                                                                                                                      [reconciled tree]
                                                                                                                      [posterior probabilities]
                                                                                                                      [speciation parameters]
                                                                                                                      [duplication parameters]
                                                                                                                      [orthostrapper/GOA]
                                                                                                                      [BLAST]
                                                                                                                      [GOtcha proteins]
                                                                                                                      [GOtcha]
                                                                                                                      [GOtcha No IEA]- PF01335: Death effector domain   
                                                                                                                        [gene tree]
                                                                                                                        [species tree]
                                                                                                                        [reconciled tree]
                                                                                                                        [posterior probabilities]
                                                                                                                        [speciation parameters]
                                                                                                                        [duplication parameters]
                                                                                                                        [orthostrapper/GOA]
                                                                                                                        [BLAST]
                                                                                                                        [GOtcha proteins]
                                                                                                                        [GOtcha]
                                                                                                                        [GOtcha No IEA]- PF01342: SAND domain   
                                                                                                                          [gene tree]
                                                                                                                          [species tree]
                                                                                                                          [reconciled tree]
                                                                                                                          [posterior probabilities]
                                                                                                                          [speciation parameters]
                                                                                                                          [duplication parameters]
                                                                                                                          [orthostrapper/GOA]
                                                                                                                          [BLAST]
                                                                                                                          [GOtcha proteins]
                                                                                                                          [GOtcha]
                                                                                                                          [GOtcha No IEA]- PF01490: Transmembrane amino acid transporter protein   
                                                                                                                            [gene tree]
                                                                                                                            [species tree]
                                                                                                                            [reconciled tree]
                                                                                                                            [posterior probabilities]
                                                                                                                            [speciation parameters]
                                                                                                                            [duplication parameters]
                                                                                                                            [orthostrapper/GOA]
                                                                                                                            [BLAST]
                                                                                                                            [GOtcha proteins]
                                                                                                                            [GOtcha]
                                                                                                                            [GOtcha No IEA]- PF01504: Phosphatidylinositol-4-phosphate 5-Kinase   
                                                                                                                              [gene tree]
                                                                                                                              [species tree]
                                                                                                                              [reconciled tree]
                                                                                                                              [posterior probabilities]
                                                                                                                              [speciation parameters]
                                                                                                                              [duplication parameters]
                                                                                                                              [orthostrapper/GOA]
                                                                                                                              [BLAST]
                                                                                                                              [GOtcha proteins]
                                                                                                                              [GOtcha]
                                                                                                                              [GOtcha No IEA]- PF01545: Cation efflux family   
                                                                                                                                [gene tree]
                                                                                                                                [species tree]
                                                                                                                                [reconciled tree]
                                                                                                                                [posterior probabilities]
                                                                                                                                [speciation parameters]
                                                                                                                                [duplication parameters]
                                                                                                                                [orthostrapper/GOA]
                                                                                                                                [BLAST]
                                                                                                                                [GOtcha proteins]
                                                                                                                                [GOtcha]
                                                                                                                                [GOtcha No IEA]- PF01553: Acyltransferase   
                                                                                                                                  [gene tree]
                                                                                                                                  [species tree]
                                                                                                                                  [reconciled tree]
                                                                                                                                  [posterior probabilities]
                                                                                                                                  [speciation parameters]
                                                                                                                                  [duplication parameters]
                                                                                                                                  [orthostrapper/GOA]
                                                                                                                                  [BLAST]
                                                                                                                                  [GOtcha proteins]
                                                                                                                                  [GOtcha]
                                                                                                                                  [GOtcha No IEA]- PF01569: PAP2 (enzyme type 2 phosphatidic acid phosphatase) superfamily   
                                                                                                                                    [gene tree]
                                                                                                                                    [species tree]
                                                                                                                                    [reconciled tree]
                                                                                                                                    [posterior probabilities]
                                                                                                                                    [speciation parameters]
                                                                                                                                    [duplication parameters]
                                                                                                                                    [orthostrapper/GOA]
                                                                                                                                    [BLAST]
                                                                                                                                    [GOtcha proteins]
                                                                                                                                    [GOtcha]
                                                                                                                                    [GOtcha No IEA]- PF01608: I/LWEQ domain   
                                                                                                                                      [gene tree]
                                                                                                                                      [species tree]
                                                                                                                                      [reconciled tree]
                                                                                                                                      [posterior probabilities]
                                                                                                                                      [speciation parameters]
                                                                                                                                      [duplication parameters]
                                                                                                                                      [orthostrapper/GOA]
                                                                                                                                      [BLAST]
                                                                                                                                      [GOtcha proteins]
                                                                                                                                      [GOtcha]
                                                                                                                                      [GOtcha No IEA]- PF01633: Choline/ethanolamine kinase   
                                                                                                                                        [gene tree]
                                                                                                                                        [species tree]
                                                                                                                                        [reconciled tree]
                                                                                                                                        [posterior probabilities]
                                                                                                                                        [speciation parameters]
                                                                                                                                        [duplication parameters]
                                                                                                                                        [orthostrapper/GOA]
                                                                                                                                        [BLAST]
                                                                                                                                        [GOtcha proteins]
                                                                                                                                        [GOtcha]
                                                                                                                                        [GOtcha No IEA]- PF01735: Lysophospholipase catalytic domain   
                                                                                                                                          [gene tree]
                                                                                                                                          [species tree]
                                                                                                                                          [reconciled tree]
                                                                                                                                          [posterior probabilities]
                                                                                                                                          [speciation parameters]
                                                                                                                                          [duplication parameters]
                                                                                                                                          [orthostrapper/GOA]
                                                                                                                                          [BLAST]
                                                                                                                                          [GOtcha proteins]
                                                                                                                                          [GOtcha]
                                                                                                                                          [GOtcha No IEA]- PF01762: Galactosyltransferase   
                                                                                                                                            [gene tree]
                                                                                                                                            [species tree]
                                                                                                                                            [reconciled tree]
                                                                                                                                            [posterior probabilities]
                                                                                                                                            [speciation parameters]
                                                                                                                                            [duplication parameters]
                                                                                                                                            [orthostrapper/GOA]
                                                                                                                                            [BLAST]
                                                                                                                                            [GOtcha proteins]
                                                                                                                                            [GOtcha]
                                                                                                                                            [GOtcha No IEA]- PF01833: IPT/TIG domain   
                                                                                                                                              [gene tree]
                                                                                                                                              [species tree]
                                                                                                                                              [reconciled tree]
                                                                                                                                              [posterior probabilities]
                                                                                                                                              [speciation parameters]
                                                                                                                                              [duplication parameters]
                                                                                                                                              [orthostrapper/GOA]
                                                                                                                                              [BLAST]
                                                                                                                                              [GOtcha proteins]
                                                                                                                                              [GOtcha]
                                                                                                                                              [GOtcha No IEA]- PF01851: Proteasome/cyclosome repeat   
                                                                                                                                                [gene tree]
                                                                                                                                                [species tree]
                                                                                                                                                [reconciled tree]
                                                                                                                                                [posterior probabilities]
                                                                                                                                                [speciation parameters]
                                                                                                                                                [duplication parameters]
                                                                                                                                                [orthostrapper/GOA]
                                                                                                                                                [BLAST]
                                                                                                                                                [GOtcha proteins]
                                                                                                                                                [GOtcha]
                                                                                                                                                [GOtcha No IEA]- PF01900: Rpp14 family   
                                                                                                                                                  [gene tree]
                                                                                                                                                  [species tree]
                                                                                                                                                  [reconciled tree]
                                                                                                                                                  [posterior probabilities]
                                                                                                                                                  [speciation parameters]
                                                                                                                                                  [duplication parameters]
                                                                                                                                                  [orthostrapper/GOA]
                                                                                                                                                  [BLAST]
                                                                                                                                                  [GOtcha proteins]
                                                                                                                                                  [GOtcha]
                                                                                                                                                  [GOtcha No IEA]- PF02173: pKID domain   
                                                                                                                                                    [gene tree]
                                                                                                                                                    [species tree]
                                                                                                                                                    [reconciled tree]
                                                                                                                                                    [posterior probabilities]
                                                                                                                                                    [speciation parameters]
                                                                                                                                                    [duplication parameters]
                                                                                                                                                    [orthostrapper/GOA]
                                                                                                                                                    [BLAST]
                                                                                                                                                    [GOtcha proteins]
                                                                                                                                                    [GOtcha]
                                                                                                                                                    [GOtcha No IEA]- PF02178: AT hook motif   
                                                                                                                                                      [gene tree]
                                                                                                                                                      [species tree]
                                                                                                                                                      [reconciled tree]
                                                                                                                                                      [posterior probabilities]
                                                                                                                                                      [speciation parameters]
                                                                                                                                                      [duplication parameters]
                                                                                                                                                      [orthostrapper/GOA]
                                                                                                                                                      [BLAST]
                                                                                                                                                      [GOtcha proteins]
                                                                                                                                                      [GOtcha]
                                                                                                                                                      [GOtcha No IEA]- PF02197: Regulatory subunit of type II PKA R-subunit   
                                                                                                                                                        [gene tree]
                                                                                                                                                        [species tree]
                                                                                                                                                        [reconciled tree]
                                                                                                                                                        [posterior probabilities]
                                                                                                                                                        [speciation parameters]
                                                                                                                                                        [duplication parameters]
                                                                                                                                                        [orthostrapper/GOA]
                                                                                                                                                        [BLAST]
                                                                                                                                                        [GOtcha proteins]
                                                                                                                                                        [GOtcha]
                                                                                                                                                        [GOtcha No IEA]- PF02319: E2F/DP family winged-helix DNA-binding domain   
                                                                                                                                                          [gene tree]
                                                                                                                                                          [species tree]
                                                                                                                                                          [reconciled tree]
                                                                                                                                                          [posterior probabilities]
                                                                                                                                                          [speciation parameters]
                                                                                                                                                          [duplication parameters]
                                                                                                                                                          [orthostrapper/GOA]
                                                                                                                                                          [BLAST]
                                                                                                                                                          [GOtcha proteins]
                                                                                                                                                          [GOtcha]
                                                                                                                                                          [GOtcha No IEA]- PF02376: CUT domain   
                                                                                                                                                            [gene tree]
                                                                                                                                                            [species tree]
                                                                                                                                                            [reconciled tree]
                                                                                                                                                            [posterior probabilities]
                                                                                                                                                            [speciation parameters]
                                                                                                                                                            [duplication parameters]
                                                                                                                                                            [orthostrapper/GOA]
                                                                                                                                                            [BLAST]
                                                                                                                                                            [GOtcha proteins]
                                                                                                                                                            [GOtcha]
                                                                                                                                                            [GOtcha No IEA]- PF02786: Carbamoyl-phosphate synthase L chain, ATP binding domain   
                                                                                                                                                              [gene tree]
                                                                                                                                                              [species tree]
                                                                                                                                                              [reconciled tree]
                                                                                                                                                              [posterior probabilities]
                                                                                                                                                              [speciation parameters]
                                                                                                                                                              [duplication parameters]
                                                                                                                                                              [orthostrapper/GOA]
                                                                                                                                                              [BLAST]
                                                                                                                                                              [GOtcha proteins]
                                                                                                                                                              [GOtcha]
                                                                                                                                                              [GOtcha No IEA]- PF02845: CUE domain   
                                                                                                                                                                [gene tree]
                                                                                                                                                                [species tree]
                                                                                                                                                                [reconciled tree]
                                                                                                                                                                [posterior probabilities]
                                                                                                                                                                [speciation parameters]
                                                                                                                                                                [duplication parameters]
                                                                                                                                                                [orthostrapper/GOA]
                                                                                                                                                                [BLAST]
                                                                                                                                                                [GOtcha proteins]
                                                                                                                                                                [GOtcha]
                                                                                                                                                                [GOtcha No IEA]- PF02864: STAT protein, DNA binding domain   
                                                                                                                                                                  [gene tree]
                                                                                                                                                                  [species tree]
                                                                                                                                                                  [reconciled tree]
                                                                                                                                                                  [posterior probabilities]
                                                                                                                                                                  [speciation parameters]
                                                                                                                                                                  [duplication parameters]
                                                                                                                                                                  [orthostrapper/GOA]
                                                                                                                                                                  [BLAST]
                                                                                                                                                                  [GOtcha proteins]
                                                                                                                                                                  [GOtcha]
                                                                                                                                                                  [GOtcha No IEA]- PF02891: MIZ zinc finger   
                                                                                                                                                                    [gene tree]
                                                                                                                                                                    [species tree]
                                                                                                                                                                    [reconciled tree]
                                                                                                                                                                    [posterior probabilities]
                                                                                                                                                                    [speciation parameters]
                                                                                                                                                                    [duplication parameters]
                                                                                                                                                                    [orthostrapper/GOA]
                                                                                                                                                                    [BLAST]
                                                                                                                                                                    [GOtcha proteins]
                                                                                                                                                                    [GOtcha]
                                                                                                                                                                    [GOtcha No IEA]- PF02984: Cyclin, C-terminal domain   
                                                                                                                                                                      [gene tree]
                                                                                                                                                                      [species tree]
                                                                                                                                                                      [reconciled tree]
                                                                                                                                                                      [posterior probabilities]
                                                                                                                                                                      [speciation parameters]
                                                                                                                                                                      [duplication parameters]
                                                                                                                                                                      [orthostrapper/GOA]
                                                                                                                                                                      [BLAST]
                                                                                                                                                                      [GOtcha proteins]
                                                                                                                                                                      [GOtcha]
                                                                                                                                                                      [GOtcha No IEA]- PF03104: DNA polymerase family B, exonuclease domain   
                                                                                                                                                                        [gene tree]
                                                                                                                                                                        [species tree]
                                                                                                                                                                        [reconciled tree]
                                                                                                                                                                        [posterior probabilities]
                                                                                                                                                                        [speciation parameters]
                                                                                                                                                                        [duplication parameters]
                                                                                                                                                                        [orthostrapper/GOA]
                                                                                                                                                                        [BLAST]
                                                                                                                                                                        [GOtcha proteins]
                                                                                                                                                                        [GOtcha]
                                                                                                                                                                        [GOtcha No IEA]- PF03131: bZIP Maf transcription factor   
                                                                                                                                                                          [gene tree]
                                                                                                                                                                          [species tree]
                                                                                                                                                                          [reconciled tree]
                                                                                                                                                                          [posterior probabilities]
                                                                                                                                                                          [speciation parameters]
                                                                                                                                                                          [duplication parameters]
                                                                                                                                                                          [orthostrapper/GOA]
                                                                                                                                                                          [BLAST]
                                                                                                                                                                          [GOtcha proteins]
                                                                                                                                                                          [GOtcha]
                                                                                                                                                                          [GOtcha No IEA]- PF03185: Calcium-activated potassium channel, beta subunit   
                                                                                                                                                                            [gene tree]
                                                                                                                                                                            [species tree]
                                                                                                                                                                            [reconciled tree]
                                                                                                                                                                            [posterior probabilities]
                                                                                                                                                                            [speciation parameters]
                                                                                                                                                                            [duplication parameters]
                                                                                                                                                                            [orthostrapper/GOA]
                                                                                                                                                                            [BLAST]
                                                                                                                                                                            [GOtcha proteins]
                                                                                                                                                                            [GOtcha]
                                                                                                                                                                            [GOtcha No IEA]- PF03234: Cdc37 family   
                                                                                                                                                                              [gene tree]
                                                                                                                                                                              [species tree]
                                                                                                                                                                              [reconciled tree]
                                                                                                                                                                              [posterior probabilities]
                                                                                                                                                                              [speciation parameters]
                                                                                                                                                                              [duplication parameters]
                                                                                                                                                                              [orthostrapper/GOA]
                                                                                                                                                                              [BLAST]
                                                                                                                                                                              [GOtcha proteins]
                                                                                                                                                                              [GOtcha]
                                                                                                                                                                              [GOtcha No IEA]- PF03253: Urea transporter   
                                                                                                                                                                                [gene tree]
                                                                                                                                                                                [species tree]
                                                                                                                                                                                [reconciled tree]
                                                                                                                                                                                [posterior probabilities]
                                                                                                                                                                                [speciation parameters]
                                                                                                                                                                                [duplication parameters]
                                                                                                                                                                                [orthostrapper/GOA]
                                                                                                                                                                                [BLAST]
                                                                                                                                                                                [GOtcha proteins]
                                                                                                                                                                                [GOtcha]
                                                                                                                                                                                [GOtcha No IEA]- PF03299: Transcription factor AP-2   
                                                                                                                                                                                  [gene tree]
                                                                                                                                                                                  [species tree]
                                                                                                                                                                                  [reconciled tree]
                                                                                                                                                                                  [posterior probabilities]
                                                                                                                                                                                  [speciation parameters]
                                                                                                                                                                                  [duplication parameters]
                                                                                                                                                                                  [orthostrapper/GOA]
                                                                                                                                                                                  [BLAST]
                                                                                                                                                                                  [GOtcha proteins]
                                                                                                                                                                                  [GOtcha]
                                                                                                                                                                                  [GOtcha No IEA]- PF03727: Hexokinase   
                                                                                                                                                                                    [gene tree]
                                                                                                                                                                                    [species tree]
                                                                                                                                                                                    [reconciled tree]
                                                                                                                                                                                    [posterior probabilities]
                                                                                                                                                                                    [speciation parameters]
                                                                                                                                                                                    [duplication parameters]
                                                                                                                                                                                    [orthostrapper/GOA]
                                                                                                                                                                                    [BLAST]
                                                                                                                                                                                    [GOtcha proteins]
                                                                                                                                                                                    [GOtcha]
                                                                                                                                                                                    [GOtcha No IEA]- PF03810: Importin-beta N-terminal domain   
                                                                                                                                                                                      [gene tree]
                                                                                                                                                                                      [species tree]
                                                                                                                                                                                      [reconciled tree]
                                                                                                                                                                                      [posterior probabilities]
                                                                                                                                                                                      [speciation parameters]
                                                                                                                                                                                      [duplication parameters]
                                                                                                                                                                                      [orthostrapper/GOA]
                                                                                                                                                                                      [BLAST]
                                                                                                                                                                                      [GOtcha proteins]
                                                                                                                                                                                      [GOtcha]
                                                                                                                                                                                      [GOtcha No IEA]- PF03982: Diacylglycerol acyltransferase   
                                                                                                                                                                                        [gene tree]
                                                                                                                                                                                        [species tree]
                                                                                                                                                                                        [reconciled tree]
                                                                                                                                                                                        [posterior probabilities]
                                                                                                                                                                                        [speciation parameters]
                                                                                                                                                                                        [duplication parameters]
                                                                                                                                                                                        [orthostrapper/GOA]
                                                                                                                                                                                        [BLAST]
                                                                                                                                                                                        [GOtcha proteins]
                                                                                                                                                                                        [GOtcha]
                                                                                                                                                                                        [GOtcha No IEA]- PF04089: BRICHOS domain   
                                                                                                                                                                                          [gene tree]
                                                                                                                                                                                          [species tree]
                                                                                                                                                                                          [reconciled tree]
                                                                                                                                                                                          [posterior probabilities]
                                                                                                                                                                                          [speciation parameters]
                                                                                                                                                                                          [duplication parameters]
                                                                                                                                                                                          [orthostrapper/GOA]
                                                                                                                                                                                          [BLAST]
                                                                                                                                                                                          [GOtcha proteins]
                                                                                                                                                                                          [GOtcha]
                                                                                                                                                                                          [GOtcha No IEA]- PF04516: CP2 transcription factor   
                                                                                                                                                                                            [gene tree]
                                                                                                                                                                                            [species tree]
                                                                                                                                                                                            [reconciled tree]
                                                                                                                                                                                            [posterior probabilities]
                                                                                                                                                                                            [speciation parameters]
                                                                                                                                                                                            [duplication parameters]
                                                                                                                                                                                            [orthostrapper/GOA]
                                                                                                                                                                                            [BLAST]
                                                                                                                                                                                            [GOtcha proteins]
                                                                                                                                                                                            [GOtcha]
                                                                                                                                                                                            [GOtcha No IEA]- PF04775: Acyl-CoA thioester hydrolase / Bile acid-CoA amino acid N-acetyltransferase   
                                                                                                                                                                                              [gene tree]
                                                                                                                                                                                              [species tree]
                                                                                                                                                                                              [reconciled tree]
                                                                                                                                                                                              [posterior probabilities]
                                                                                                                                                                                              [speciation parameters]
                                                                                                                                                                                              [duplication parameters]
                                                                                                                                                                                              [orthostrapper/GOA]
                                                                                                                                                                                              [BLAST]
                                                                                                                                                                                              [GOtcha proteins]
                                                                                                                                                                                              [GOtcha]
                                                                                                                                                                                              [GOtcha No IEA]- PF05349: GATA-type transcription activator, N-terminal   
                                                                                                                                                                                                [gene tree]
                                                                                                                                                                                                [species tree]
                                                                                                                                                                                                [reconciled tree]
                                                                                                                                                                                                [posterior probabilities]
                                                                                                                                                                                                [speciation parameters]
                                                                                                                                                                                                [duplication parameters]
                                                                                                                                                                                                [orthostrapper/GOA]
                                                                                                                                                                                                [BLAST]
                                                                                                                                                                                                [GOtcha proteins]
                                                                                                                                                                                                [GOtcha]
                                                                                                                                                                                                [GOtcha No IEA]- PF06009: Laminin Domain II   
                                                                                                                                                                                                  [gene tree]
                                                                                                                                                                                                  [species tree]
                                                                                                                                                                                                  [reconciled tree]
                                                                                                                                                                                                  [posterior probabilities]
                                                                                                                                                                                                  [speciation parameters]
                                                                                                                                                                                                  [duplication parameters]
                                                                                                                                                                                                  [orthostrapper/GOA]
                                                                                                                                                                                                  [BLAST]
                                                                                                                                                                                                  [GOtcha proteins]
                                                                                                                                                                                                  [GOtcha]
                                                                                                                                                                                                  [GOtcha No IEA]- PF06068: TIP49 C-terminus   
                                                                                                                                                                                                    [gene tree]
                                                                                                                                                                                                    [species tree]
                                                                                                                                                                                                    [reconciled tree]
                                                                                                                                                                                                    [posterior probabilities]
                                                                                                                                                                                                    [speciation parameters]
                                                                                                                                                                                                    [duplication parameters]
                                                                                                                                                                                                    [orthostrapper/GOA]
                                                                                                                                                                                                    [BLAST]
                                                                                                                                                                                                    [GOtcha proteins]
                                                                                                                                                                                                    [GOtcha]
                                                                                                                                                                                                    [GOtcha No IEA]- PF06464: DMAP1-binding Domain   
                                                                                                                                                                                                      [gene tree]
                                                                                                                                                                                                      [species tree]
                                                                                                                                                                                                      [reconciled tree]
                                                                                                                                                                                                      [posterior probabilities]
                                                                                                                                                                                                      [speciation parameters]
                                                                                                                                                                                                      [duplication parameters]
                                                                                                                                                                                                      [orthostrapper/GOA]
                                                                                                                                                                                                      [BLAST]
                                                                                                                                                                                                      [GOtcha proteins]
                                                                                                                                                                                                      [GOtcha]
                                                                                                                                                                                                      [GOtcha No IEA]- PF07392: Cyclin-dependent kinase inhibitor 2a p19Arf N-terminus   
                                                                                                                                                                                                        [gene tree]
                                                                                                                                                                                                        [species tree]
                                                                                                                                                                                                        [reconciled tree]
                                                                                                                                                                                                        [posterior probabilities]
                                                                                                                                                                                                        [speciation parameters]
                                                                                                                                                                                                        [duplication parameters]
                                                                                                                                                                                                        [orthostrapper/GOA]
                                                                                                                                                                                                        [BLAST]
                                                                                                                                                                                                        [GOtcha proteins]
                                                                                                                                                                                                        [GOtcha]
                                                                                                                                                                                                        [GOtcha No IEA]

---

Barbara Engelhardt


Last modified: Mon Dec 6 10:17:26 PST 2004
