# Supplementary material for: Protein Molecular Function Prediction by Bayesian Phylogenomics
Source: PLoS Comput Biol. 2005 Oct 7;1(5):e45. doi: 10.1371/journal.pcbi.0010045 (PMC1246806; doi:10.1371/journal.pcbi.0010045)
Supplement: Supplementary file 1 [file pcbi.0010045.sd001.tgz › sifter/deaminase.html]

Results for AMP/Adenosine Deaminase


# Results for AMP/Adenosine Deaminase

For each file, the columns are explained in this README.
  

|  |  |
| --- | --- |
| Posterior probabilities for the family when experimental evidence from GO annotation database is included. Used to create ROC plot on right.   PubMed links for each protein associated with an experimental function assay.   BLAST E-values for the family, for the first occurrence of each of the labels adenosine/adenine/AMP/growth factor activity. Used to create ROC plot on right. |  |

---

**Kinetics analysis for Q9IJA8\_PLAFA**
  

|  |  |
| --- | --- |
| Adenosine deaminase results for Q9IJA8\_PLAFA, showing adenosine deaminase activity. The kcat is 9.3 +/- 0.5 s-1, and the Km is 11 +/- 2 M.   Adenine deaminase results for Q9IJA8\_PLAFA, showing adenine deaminase activity. There is no detectable activity across the range of concentrations for this substrate.   AMP deaminase results for Q9IJA8\_PLAFA, showing AMP deaminase activity. There is no detectable activity across the range of concentrations for this substrate. |  |

  


---

**Phylogeny tree including posterior probability results**
  


---

Barbara Engelhardt


Last modified: Mon Dec 6 10:21:07 PST 2004
