# Supplementary material for: Protein Molecular Function Prediction by Bayesian Phylogenomics
Source: PLoS Comput Biol. 2005 Oct 7;1(5):e45. doi: 10.1371/journal.pcbi.0010045 (PMC1246806; doi:10.1371/journal.pcbi.0010045)
Supplement: Supplementary file 1 [file pcbi.0010045.sd001.tgz › sifter/dehydrogenase.html]

Results for Lactate/Malate Dehydrogenase


# Results for Lactate/Malate Dehydrogenase

For each file, the columns are explained in this README.
  

|  |  |
| --- | --- |
| Posterior probabilities for the family when experimental evidence from GO annotation database is included. Used to make ROC plot on right.   PubMed links for each protein associated with an experimental function assay.   Top BLAST hits for the family, for differentiating MDH from LDH and differentiating the two substrates and the two cofactors.   BLAST E-values for the family, for the first occurrence of each of the labels LDH/MDH and each of the labels with substrate and cofactor L-LDH/MDH NAD(+)/MDH NADP(+). Used to make ROC plot on right.   GeneQuiz results for the family. |  |

---


---

Barbara Engelhardt


Last modified: Tue Nov 30 11:41:15 PST 2004
