# Supplementary material for: Protein Molecular Function Prediction by Bayesian Phylogenomics
Source: PLoS Comput Biol. 2005 Oct 7;1(5):e45. doi: 10.1371/journal.pcbi.0010045 (PMC1246806; doi:10.1371/journal.pcbi.0010045)
Supplement: Supplementary file 1 [file pcbi.0010045.sd001.tgz › sifter/pf00056-pubmed.html]

DHL2\_LACCO: 7643402  
LDH1\_BIFLO: 2695396  
LDH1\_LACLA: 1400245  
LDH1\_LACPL: 8300514  
LDH1\_PLAFD: 8515777  
LDH2\_BIFLO: 8450537  
LDH2\_LACLA: 2330370  
LDH2\_LACPL: 2330370  
LDHA\_ALLMI: 9364765  
LDHA\_COLLI: 9364765  
LDHA\_HUMAN: 11276087  
LDHA\_MOUSE: 9457676  
LDHA\_PIG: 1678537  
LDHA\_RABIT: 2917988  
LDHA\_RAT: 9600066  
LDHA\_RHIOR: 10831409  
LDHA\_SCEWO: 8666293  
LDHA\_SPHAG: 9115998  
LDHA\_SPHID: 9115998  
LDHA\_SPHLU: 9115998  
LDHA\_SQUAC: 197516  
LDHA\_TRASC: 9364765  
LDHA\_XENLA: 7937776  
LDHB\_ALLMI: 9364765  
LDHB\_ANAPL: 89017148  
LDHB\_CAICA: 11722846  
LDHB\_COLLI: 9364765  
LDHB\_FUNHE: 2615640  
LDHB\_FUNPA: 8105474  
LDHB\_HUMAN: 3435492  
LDHB\_MOUSE: 2338075  
LDHB\_PELSJ: 1172846  
LDHB\_PIG: 7937776  
LDHB\_RABIT: 2917988  
LDHB\_RAT: 1582561  
LDHB\_RHIOR: 10831409  
LDHB\_SCEUN: 10508547  
LDHB\_SCEWO: 8666293  
LDHB\_SQUAC: 9628010  
LDHB\_TRASC: 9364765  
LDHB\_XENLA: 7937776  
LDHC\_FUNHE: 8419929  
LDHC\_HUMAN: 2440048  
LDHC\_MOUSE: 468772  
LDHC\_RAT: 7937776  
LDHC\_XENLA: 7937776  
LDHP\_BACPS: 2334516  
LDHX\_BACPS: 2334516  
LDH\_BACCA: 3765869, 3297694  
LDH\_BACCL: 3765869  
LDH\_BACME: 3118900, 3122782  
LDH\_BACST: 1731077, 6352452  
LDH\_BACSU: 3098260  
LDH\_CAEEL: 7999079  
LDH\_DEIRA: 8654970  
LDH\_DROME: 9094207  
LDH\_LACCA: 6411465  
LDH\_LACLC: 8081494  
LDH\_LACPE: 1425707  
LDH\_LACSK: 99098693  
LDH\_MYCGA: 1627781  
LDH\_MYCHY: 7679720  
LDH\_PEDAC: 7887607  
LDH\_PETMA: 1542673  
LDH\_STRBO: 9142744  
LDH\_STRMU: 1937751  
LDH\_STRPN: 9662606  
LDH\_THECA: 2185236  
LDH\_THEMA: 9655830  
LDH\_TOXGO: 14744130  
MDHC\_MESCR: 9358050  
MDHC\_MOUSE: 3172222  
MDHC\_PIG: 8682322  
MDHC\_YEAST: 1986231  
MDHG\_ARATH: 9774405  
MDHG\_CITLA: 2377615  
MDHG\_CUCSA: 2858221  
MDHG\_SOYBN: 8547819  
MDHM\_ARATH: 9774405  
MDHM\_CITLA: 2102869  
MDHM\_EUCGU: 7770532  
MDHM\_PIG: 8117664  
MDHM\_RAT: 3755817  
MDHM\_SCHMA: 8396206  
MDHM\_YEAST: 3072021  
MDHP\_FLABI: 10196131  
MDHP\_MAIZE: 3403553  
MDHP\_MEDSA: 9721676  
MDHP\_PEA: 8223554  
MDHP\_SORBI: 10194350  
MDHP\_YEAST: 1447211  
MDHQ\_SORBI: 1896015  
MDH\_AQUAR: 10206992  
MDH\_ARCFU: 9211715, 11513609  
MDH\_BACIS: 8694770  
MDH\_BACSU: 8550482  
MDH\_BURPS: 9190829  
MDH\_CHLAU: 8661927  
MDH\_CHLTE: 12054817, 1735722  
MDH\_CHLVI: 1735722  
MDH\_ECOLI: 1507230  
MDH\_HALMA: 12581646  
MDH\_METJA: 10998181  
MDH\_NITAL: 2930509  
MDH\_PHEIM: 2775496  
MDH\_STRAR: 2775496  
MDH\_THETH: 8471609  
MDH\_VIBS5: 8998993  
MDH\_XANMA: 9190829  
Q9Y750: 10103045  
O96518: 10339579  
P90613: 9016946  
Q9NJT1: 10583384  
O15769: 9603490  
O96445: 10339579  
Q9GT30: 10900211  
Q27819: 8595866  
Q9GT92: 10900211  
Q9NHX3: 10713448  
Q9NHX2: 10713448  
Q9Y1U1: 10229579  
Q9GQB4: 11110897  
O44340: 9402738  
Q95WV4: 11606232  
O76731: 9851617  
Q9GCV9: 11111052  
Q9SAN7: 9426613  
Q9FNS5: 11855723  
Q9FNR7: 11111052  
Q43830: 2209586  
O48904: 9721676  
Q96570: 9426593  
O48903: 9721676  
O48906: 9721676  
Q42737: 7630939  
Q59843: 8108402  
Q99PZ5: 10024551  
Q9R868: 9662606  
Q8RJW4: 9190829  
O34615: 9199437  
Q9FDQ5: 1507230  
Q60159: 8108402  
Q59842: 8108402  
Q60166: 8108402  
Q933J3: 1507230  
Q60130: 8108402  
Q93R02: 1507230  
Q9AG50: 10024551  
Q9R3N3: 10024551  
Q59840: 8108402  
Q59845: 8108402  
Q59841: 8108402  
Q9FDQ3: 1507230  
Q9AG49: 10024551  
Q9AIU2: 10024551  
Q59838: 8108402  
Q60156: 8108402  
Q9R3N2: 10024551  
Q59839: 8108402  
Q8VVY0: 9662606  
Q8VLI8: 9662606  
Q60155: 8108402  
Q9FDQ4: 1507230  
Q99Q77: 10024551  
Q59846: 8108402  
Q9XCZ8: 10024551  
Q9S4R7: 10024551  
Q59844: 8108402  
P94886: 8081494  
Q99QN5: 10024551  
Q8GN37: 10024551  
Q8GMW6: 8300514  
Q64483: 3619944  
Q8UUQ5: 9364765  
Q9DDB1: 11251784  
Q9DDB2: 11251784  
Q801E9: 12612830  
Q801E8: 12612830  
Q801E7: 12612830  
Q801E6: 12612830  
Q7ZSY2: 12612830
