# Supplementary material for: Protein Molecular Function Prediction by Bayesian Phylogenomics
Source: PLoS Comput Biol. 2005 Oct 7;1(5):e45. doi: 10.1371/journal.pcbi.0010045 (PMC1246806; doi:10.1371/journal.pcbi.0010045)
Supplement: Supplementary file 1 [file pcbi.0010045.sd001.tgz › sifter/pf00962-pubmed.html]

ADA\_BOVIN: 9977857, 11067872  
ADA\_HUMAN: 3839456  
ADA\_MOUSE: 2387582  
ADA\_YEAST: 6392474  
ADD\_ECOLI: 1998686  
ADD\_ECO57: 1998686  
ADD\_ECOL6: 1998686  
ADD\_STRVG: 8675024  
AGSA\_APLCA: 2777814  
AMD1\_HUMAN: 1370861  
AMD1\_RAT: 3624265  
AMD2\_HUMAN: 8764830  
AMD2\_RAT: 2365682  
AMD3\_HUMAN: 8004104  
AMD3\_MOUSE: 9133604  
AMD3\_RAT: 9291127  
AMDM\_YEAST: 8241153  
Q8X1T6: 14643670  
Q9VFS0: 11738815  
Q9VFS1: 11738815  
Q9U7C5: 10903440  
Q9NC65: 10864517  
Q26642: 8662793  
Q9U7C6: 10903440  
Q9GRE6: 10967093  
Q9P6I7: 14643670  
Q9V741: 1178815  
Q9VVK5: 11738815
