# Supplementary material for: Protein Molecular Function Prediction by Bayesian Phylogenomics
Source: PLoS Comput Biol. 2005 Oct 7;1(5):e45. doi: 10.1371/journal.pcbi.0010045 (PMC1246806; doi:10.1371/journal.pcbi.0010045)
Supplement: Supplementary file 1 [file pcbi.0010045.sd001.tgz › sifter/resampled.html]

Resampled Analyses


# Resampled Analyses

## Overall Results

File formats

|  |  |
| --- | --- |
| - Posterior probabilities- Errors- Error       frequency, by protein |  |

---

## Alignment Methods

- ClustalW [posteriors|errors|by protein], with options:  
  BLOSUM  
   BLOSUM, fast  
  PAM  
  PAM, fast  
  - MAFFT [posteriors|errors|by protein], with options:  
    FFT-NS-1: alignment
    distance matrix  
    FFT-NS-2: alignment
    distance matrix  
    FFT-NS-3: alignment
    distance matrix  
    FFT-NS-4: alignment
    distance matrix  
    FFT-NS-5: alignment
    distance matrix  
    NW-NS-1: alignment
    distance matrix  
    NW-NS-2: alignment
    distance matrix  
    NW-NS-3: alignment
    distance matrix  
    NW-NS-4: alignment
    distance matrix  
    NW-NS-5: alignment
    distance matrix  
    - MUSCLE [posteriors|errors|by protein]  
      Default: alignment
      distance matrix

---

## Phylogeny Reconstruction Methods

- Neighbor Joining (a distance method) [posteriors|errors|by protein]  
  ClustalW (BLOSUM): reconciled tree
  output A
  output B   
  ClustalW (BLOSUM, fast): reconciled tree
  output A
  output B   
  ClustalW (PAM): reconciled tree
  output A
  output B   
  ClustalW (PAM, fast): reconciled tree
  output A
  output B   
  MAFFT (FFT1): reconciled tree
  output A
  output B   
  MAFFT (FFT2): reconciled tree
  output A
  output B   
  MAFFT (FFT3): reconciled tree
  output A
  output B   
  MAFFT (FFT4): reconciled tree
  output A
  output B   
  MAFFT (FFT5): reconciled tree
  output A
  output B   
  MAFFT (NW1): reconciled tree
  output A
  output B   
  MAFFT (NW2): reconciled tree
  output A
  output B   
  MAFFT (NW3): reconciled tree
  output A
  output B   
  MAFFT (NW4): reconciled tree
  output A
  output B   
  MAFFT (NW5): reconciled tree
  output A
  output B   
  MUSCLE: reconciled tree
  output A
  output B   
    
  - UPGMA (a distance method) [posteriors|errors|by protein]  
    ClustalW (BLOSUM): reconciled tree
    output A
    output B   
    ClustalW (BLOSUM, fast): reconciled tree
    output A
    output B   
    ClustalW (PAM): reconciled tree
    output A
    output B   
    ClustalW (PAM, fast): reconciled tree
    output A
    output B   
    MAFFT (FFT1): reconciled tree
    output A
    output B   
    MAFFT (FFT2): reconciled tree
    output A
    output B   
    MAFFT (FFT3): reconciled tree
    output A
    output B   
    MAFFT (FFT4): reconciled tree
    output A
    output B   
    MAFFT (FFT5): reconciled tree
    output A
    output B   
    MAFFT (NW1): reconciled tree
    output A
    output B   
    MAFFT (NW2): reconciled tree
    output A
    output B   
    MAFFT (NW3): reconciled tree
    output A
    output B   
    MAFFT (NW4): reconciled tree
    output A
    output B   
    MAFFT (NW5): reconciled tree
    output A
    output B   
    MUSCLE: reconciled tree
    output A
    output B   
      
    - Fitch-Margoliash (a distance method) [posteriors|errors|by protein]  
      ClustalW (PAM, fast): reconciled tree
      output A
      output B   
      ClustalW (PAM, fast): reconciled tree
      output A
      output B   
      MAFFT (FFT1): reconciled tree
      output A
      output B   
      MAFFT (FFT2): reconciled tree
      output A
      output B   
      MAFFT (FFT4): reconciled tree
      output A
      output B   
      MAFFT (FFT5): reconciled tree
      output A
      output B   
      MAFFT (NW1): reconciled tree
      output A
      output B   
      MAFFT (NW2): reconciled tree
      output A
      output B   
      MAFFT (NW3): reconciled tree
      output A
      output B   
      MAFFT (NW4): reconciled tree
      output A
      output B   
      MAFFT (NW5): reconciled tree
      output A
      output B   
      MUSCLE: reconciled tree
      output A
      output B   
        
      - Parsimony (no distances, see reconciled trees) [posteriors|errors|by protein]  
        ClustalW (BLOSUM): reconciled tree
        output A
        output B   
        ClustalW (BLOSUM): reconciled tree
        output A
        output B   
        ClustalW (BLOSUM): reconciled tree
        output A
        output B   
        ClustalW (PAM): reconciled tree
        output A
        output B   
        ClustalW (PAM): reconciled tree
        output A
        output B   
        ClustalW (PAM): reconciled tree
        output A
        output B   
        ClustalW (PAM, fast): reconciled tree
        output A
        output B   
        MAFFT (FFT1): reconciled tree
        output A
        output B   
        MAFFT (FFT1): reconciled tree
        output A
        output B   
        MAFFT (FFT2): reconciled tree
        output A
        output B   
        MAFFT (FFT2): reconciled tree
        output A
        output B   
        MAFFT (FFT3): reconciled tree
        output A
        output B   
        MAFFT (FFT4): reconciled tree
        output A
        output B   
        MAFFT (FFT4): reconciled tree
        output A
        output B   
        MAFFT (FFT5): reconciled tree
        output A
        output B   
        MAFFT (FFT5): reconciled tree
        output A
        output B   
        MAFFT (NW1): reconciled tree
        output A
        output B   
        MAFFT (NW1): reconciled tree
        output A
        output B   
        MAFFT (NW2): reconciled tree
        output A
        output B   
        MAFFT (NW2): reconciled tree
        output A
        output B   
        MAFFT (NW3): reconciled tree
        output A
        output B   
        MAFFT (NW4): reconciled tree
        output A
        output B   
        MAFFT (NW4): reconciled tree
        output A
        output B   
        MAFFT (NW5): reconciled tree
        output A
        output B   
        MAFFT (NW5): reconciled tree
        output A
        output B   
        MUSCLE: reconciled tree
        output A
        output B   
          
        - Maximum Likelihood   
          MAFFT (FFT1): reconciled tree
          output A
          output B   
          MAFFT (FFT2): reconciled tree
          output A
          output B

---

Barbara Engelhardt


Last modified: Tue Nov 30 11:31:12 PST 2004
